# Improvements in Gene Editing Technology Boost Its Applications in Livestock

**DOI:** 10.3389/fgene.2020.614688

**Published:** 2021-01-08

**Authors:** Iuri Viotti Perisse, Zhiqiang Fan, Galina N. Singina, Kenneth L. White, Irina A. Polejaeva

**Affiliations:** ^1^Department of Animal, Dairy and Veterinary Sciences, Utah State University, Logan, UT, United States; ^2^L.K. Ernst Federal Research Center for Animal Husbandry, Podolsk, Russia

**Keywords:** CRISPR/Cas9, agriculture, animal models, livestock, gene editing

## Abstract

Accelerated development of novel CRISPR/Cas9-based genome editing techniques provides a feasible approach to introduce a variety of precise modifications in the mammalian genome, including introduction of multiple edits simultaneously, efficient insertion of long DNA sequences into specific targeted loci as well as performing nucleotide transitions and transversions. Thus, the CRISPR/Cas9 tool has become the method of choice for introducing genome alterations in livestock species. The list of new CRISPR/Cas9-based genome editing tools is constantly expanding. Here, we discuss the methods developed to improve efficiency and specificity of gene editing tools as well as approaches that can be employed for gene regulation, base editing, and epigenetic modifications. Additionally, advantages and disadvantages of two primary methods used for the production of gene-edited farm animals: somatic cell nuclear transfer (SCNT or cloning) and zygote manipulations will be discussed. Furthermore, we will review agricultural and biomedical applications of gene editing technology.

## Introduction

The development of CRISPR/Cas9-based genome editing tool has revolutionized the field, and led to the modification of livestock genomes with much greater simplicity and efficiency (Urnov et al., 2010; Joung and Sander, 2013; Laible et al., 2015; Lillico et al., 2016; Georges et al., 2019). CRISPR technology was first applied to the mammalian genome in 2013 (Cong et al., 2013) and subsequently, expanded to a wide range of cell lines and mammalian species including livestock. This technology allows for modifications that lead to improvements in livestock production traits, animal health, and welfare, generation of more refined large animal models of human diseases, pharmaceutical protein production, and investigating gene function. Since 2014, over 500 research papers have been published using CRISPR gene editing approach in livestock (pigs, cattle, sheep, and goats; based on the October 1st, 2020 PubMed search).

Precise genome editing is based on the ability of engineered nucleases ZFNs (Zinc Finger Nucleases), TALENs (Transcription Activator-Like Effector Nucleases), and CRISPR (Clustered Regularly Interspaced Short Palindromic Repeats) to cut the genome in a specific targeted position. Then, the resulting double-stranded break (DSB) triggers the cell repair mechanism to repair the damage by either non-homologous end-joining (NHEJ) or homology-directed repair (HDR), which introduces a targeted mutation into a specific genomic location (McMahon et al., 2012). The CRISPR/Cas9 system is a simple and versatile method compared to ZFN and TALEN approaches that require the assembly of the associated engineered proteins for each target. The efficiency of CRISPR-based genome editing has increased to the point that the technology allows multiple edits simultaneously (Georges et al., 2019), which has led to this becoming the method of choice for introduction of specific genomic modifications in livestock species. The list of new CRISPR/Cas9-based genome editing tools is constantly expanding. This review will discuss the methods developed to improve efficiency and specificity of gene editing tools as well as approaches that can be employed for gene regulation, base editing, and epigenetic modifications. Advantages and disadvantages of two primary methods used for the production of gene-edited farm animals: somatic cell nuclear transfer (SCNT) and zygote manipulations will also be discussed. We will also review the use of gene editing technology in agriculture and biomedicine.

## Gene Editing Techniques

Several comprehensive reviews discussing gene-editing technology and its current status in livestock are available (Kalds et al., 2019; McFarlane et al., 2019; Kalds et al., 2020; Lee et al., 2020; Menchaca et al., 2020a; Navarro-Serna et al., 2020), therefore, we provide an overview of critical landmark events and recent improvements in the CRISPR/Cas9 field and include a comprehensive literature review focused on the production of gene edited farm animals with specific application to agricultural and biomedical fields.

### ZFNs

The chimeric nucleases, ZFNs, were developed in 2001 (Bibikova et al., 2001) and designed to target and disrupt precise DNA sequences (Qomi et al., 2019). Zinc fingers are small protein (20–30 amino acids) motifs regulated by zinc ion that binds to DNA, recognizing a 3-base pair (bp) sequence. The motifs have been combined with the genetically engineered restriction enzyme *Fok*I to create a programmable nuclease with the ability to identify target sequence sites. The ZFNs are effective when two zinc finger modules bind to the DNA in sites that opose each other with the *Fok*I enzyme in the middle, which forms a homodimer complex. Once the homo-dimerization is established, the nuclease breaks both DNA strands, and mutations are randomly inserted (Adli, 2018). The target site can be designed by changing the residues in a single zinc finger that alters its specificity for DNA recognition, thus, the finger motifs can be customized to recognize many different DNA triplet nucleotides (Carroll, 2017). Although ZFNs were innovative due to their higher specificity to the DNA sequence, they have a few major disadvantages, such as an exhaustive time-consuming process to design a pair of ZFNs against a target sequence. Also, there are a low number of potential targets in the genome, which makes this gene editing molecule not applicable to many studies. In fact, for every 50-bp, only one locus is suitable for this approach (Qomi et al., 2019).

### TALENs

In search of more efficient gene editing tools, in 2009, a new generation of nucleases, transcription activator-like effector nuclease emerged. Originally found in the plant pathogenic bacteria *Genus Xanthomonas*, the transcription activator-like effectors (TALEs) are DNA-binding domains containing 33–35 amino acid repeat motifs that identify each of the bps. Its site-specificity is determined by two hypervariable amino acids known as repeat-variable di-residues (Gaj et al., 2013). Similar to ZFNs, TALEs have been engineered to fuse with the DNA-cutting domain of the *Fok*I nuclease to serve as a gene editing tool known as TALENs (Adli, 2018). The difference between the ZFNs and TALENs is related to the number of nucleotides recognized by the protein domains, 3-bp versus 1-bp, thus making TALENs more site-specific and less likely to cause an off-target cleavage (Khan, 2019).

### CRISPR/Cas9

Although ZFNs and TALENs have offered vast improvements for gene manipulation, the most significant discovery came in 2013 when Dr. Zhang and colleagues successfully accomplished the first CRISPR/Cas9 genome editing in mammals (Cong et al., 2013). The unusual 29 sequence RNA repeats were initially found in 1987 by Yoshizumi Ishino at Osaka University while studying *Escherichia coli* bacteria. Years later, in 2002, the molecule was named by Drs. Mojica and Ruud Jansen as CRISPR, an abbreviation for Clustered Regularly Interspaced Short Palindromic Repeats (Mojica et al., 2000; Hsu et al., 2014). CRISPR and CRISPR-associated protein (Cas) can be easily customized to effectively introduce mutations at specific locations within genes in mammalian cells (Cong et al., 2013). The CRISPR/Cas9 complex was elucidated as a primitive acquired immune system of some bacteria and most of the archaea species to defend against the foreign DNA of bacteriophage (Humphrey and Kasinski, 2015). This mechanism consisted of two phases: immunization and immunity phases. In the immunization phase, Cas1 and Cas2 endonucleases recognize the viral genome, break it into small fragments and insert them into the bacterial genome as repeat-spacer units. During a subsequent viral invasion (immunity phase), the bacteria produce precursor-CRISPR RNA (pre-crRNA) based on the previously captured repeat-spacer units. The pre-crRNA binds to the Cas9 endonuclease and trans-activating crRNA (tracrRNA) forming the crRNA-Cas9-tracrRNA complex (Marraffini, 2015; Qomi et al., 2019). The complex is then degraded by RNase III, which results in the cleavage of each repeat fragment, turning the long CRISPR precursor into small crRNA guides for targeting the exogenous DNA. This CRISPR-Cas immunity promotes the DSB of invading DNA (Marraffini, 2015).

**FIGURE 1:**
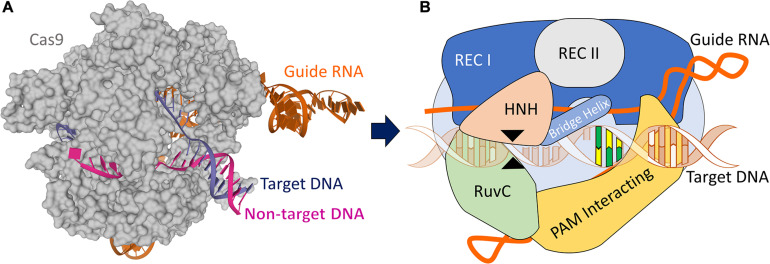
CRISPR/Cas9 structure. **(A)** X-ray structure of the Streptococcus pyogenes (Sp) CRISPR/Cas9 system (5F9R.pdb) in the pre-activated state (Jiang et al., 2016), created using Mol* (Sehnal et al., 2018). Cas9 (gray) is shown in molecular surface. The guide RNA (orange), the target DNA (dark blue), and non-target DNA (pink) strands are shown as cartoons. **(B)** A schematic CRISPR/Cas9 ribonucleoprotein structure formed by six domains: Rec I, Rec II, RuvC, HNH, Bridge Helix, and PAM Interacting domain, and guide RNA targeting DNA. The black arrow heads indicate the cut sites from each RuvC and HNH domains. The yellow/green nucleotides represent the PAM sequence.

The CRISPR/Cas9 system consists of the Cas9 endonuclease with putative nuclease and helicase domains bound to a tracrRNA:crRNA duplex. The crRNA region contains 20 customizable nucleotides at 5′ end that forms the guide RNA (gRNA) and a repeat region with 12 nucleotides, whereas the tracrRNA consists of 14 nucleotides anti-repeat region and three loops (Mei et al., 2016). The duplex RNA is responsible for guiding the Cas9 to the specific sequence on the DNA where the gRNA aligns against the complementary sequence. With the target sequence found, the helicase domain works by opening the double strands while the nuclease sites (RuvC and HNH) perform the DSB of the DNA (Figure 1). Subsequently, the crRNA:tracrRNA has been genetically engineered to become a single guide RNA with changeable 5′ nucleotides. In addition to the gRNA identification, the designed target sequence must be located upstream to a protospacer-adjacent motif (PAM) – 5′-NGG-3′ where N can be any of the four known DNA nucleotides to be recognized by the Cas9 nuclease (Yang, 2015). Experiments have shown that the Cas9 starts the target site-searching process by probing a suitable PAM sequence before matching the gRNA complementary to the DNA. The identification of the site occurs through the molecular interactions between the gRNA with the target DNA nucleotides, and once mismatched, the Cas9 rapidly dissociates from the DNA. The Cas9 only triggers the DSB after a precise complementarity between the gRNA and the target DNA have been reached, which provides the energy to the enzyme to break the DNA (Jiang and Doudna, 2017).

## DNA Repair Mechanisms: NHEJ and HDR

Genes can be effectively knocked out by merely producing mutations through a DSB of the targeted gene by engineered nucleases. After the break, the cells naturally attempt to repair the damage by using one of the two main repair mechanisms: the NHEJ and HDR pathways (Riordan et al., 2015).

The NHEJ system is the primary DNA repair mechanism for DNA DSB. It involves a straight ligation of the blunt ends, produced by the symmetric break of the DNA, using a complex of Ku70/80 proteins associated with the DNA Ligase IV (Pannunzio et al., 2018). NHEJ is the homology-independent pathway as it involves the alignment of only one to a few complementary bases for the re-ligation of two ends. It is an error-prone repair mechanism and frequently results in out-of-frame mutations (insertions or deletions – indels) in the repaired sequence. Moreover, even when an appropriate DNA repair takes place, the CRISPR/Cas9 continues to bind and disrupt the DNA sequence increasing the possibility of subsequent mutations. Indels often promote frameshift alteration of the codons, which leads to a disruption of the protein-coding sequence and often a premature stop codon (Dow, 2015). Thus, the strategy of gene inactivation by indels introduction is known as knockout (KO). The CRISPR/Cas9 tool has being successfully used in many organisms and cell types (e.g., human, sheep, goat, cattle, pig, and mouse) (Mei et al., 2016; DeWitt et al., 2017; Xie et al., 2017; Seki and Rutz, 2018; Jin et al., 2019) with efficiency ranging from 10% to over 90%. Initial use of CRISPR/Cas9 relied on plasmid transfection, but since CRISPR/Cas9 ribonucleoprotein (RNP) has become commercially available, RNP delivery provides higher KO efficiency (DeWitt et al., 2017; Perisse et al., 2020) and avoids the pitfalls associated with use of DNA plasmid delivery. The RNP provides fast action to perform DSB and indels are detectible very shortly after CRISPR/Cas9 RNP delivery. RNP is cleared from the cells within 24 h, thus, reducing the risk of off-target mutations. In contrast, plasmid delivery risks unintentional off target mutation and may also result in a vector integration into the host genome (DeWitt et al., 2017).

The second DSB repair mechanism is the HDR pathway, which uses the allelic gene from the sister chromatid as template DNA for reconstitution of the original sequence (Johnson and Jasin, 2000). The template DNA provides information to repair precisely the damaged chromosomes (Yeh et al., 2019). This repair system is highly specific and precise but in eukaryotic cells its occurrence is much lower due to the high prevalence of NHEJ (Riordan et al., 2015). The HDR takes place during synthesis (S) through G2 phases of the cell cycle (Zhao et al., 2017). When a sister chromatid is available, cyclin-dependent kinases 1 and 2 (CDK1/2) phosphorylate *C*-terminal binding protein (CtBP)-interacting protein (CtIP) endonucleases. These nucleases activate with the MRN (Mre11, Rad50, Nbs1) protein complex that binds to the damaged DNA strands (Yeh et al., 2019). Then, CtIP promotes the resection of the damaged DNA, which is crucial for homologous recombination. The resection results in a longer 3′ single-stranded DNA (ssDNA) fragments that are coated by replication protein A (RPA). This protein is replaced by Rad51 to form a nucleoprotein presynaptic filament, which facilitates the search for a homologous DNA sequence. Once the donor DNA is aligned, the new DNA strands are synthesized followed by the dissociation of Rad51 and ligation of the DNA breaks (Pawelczak et al., 2018).

## Improvements of CRISPR/Cas9

### Cas9 Nickase (nCas9)

This modified Cas9 endonuclease has been engineered to increase the efficiency of single point-mutation introduction and specificity to the target gene. The enzyme was modified to cut a single strand by either the RuvC or the HNH domain (see Figure 1B) and thus, being named Cas9 “nickase.” The nCas9 (nickase) contains one inactive domain (inactivated through one amino acid substitution in the protein-coding sequence) along with another functional domain that retains the ability to create a single-strand DNA break providing the opportunity for directed modification. By using two different gRNAs with targets that are close to each other in combination with a nCas9, a process known as “double nicking,” the gene editing based on nCas9 increases the specificity and reduces the chances of off-target mutation events without affecting the on-target efficacy (Cho et al., 2014; Adli, 2018). The CRIPSR/Cas9 recognition mechanism typically may tolerate up to three nucleotide sequence mismatches between gRNA and target DNA, though as many as six have been previously reported (Tsai et al., 2015; Tycko et al., 2016). Undesirable off-target mutations could lead to alterations in gene expression or protein function, potentially introduce genotoxicity, and reduce cell viability. It is estimated that off-target activity can be decreased by 50- to 1,500-fold in cell lines when using double nicking (Zhang X. H. et al., 2015; Harrison and Hart, 2018).

### Dead Cas9 (dCas9)

Another modified Cas9 is known as nuclease-null deactivated Cas9 or “dead Cas9.” The dCas9 is designed to prevent double or single strand DNA breaks. With RuvC and HNH (Figure 1B) nuclease domains inactive, the CRISPR/dCas9 is capable to find the target sequence and cause direct transcriptional perturbation of the gene without causing a damage in the DNA. This dCas9 can be fused with proteins in order to inhibit (CRISPRi) or activate (CRISPRa) gene expression. For instance, Cas9 fused with Kruppel-associated box (KRAB) promotes gene repression whereas the enzyme fused with VP16 or VP64 activates gene expression (Gilbert et al., 2013; Lawhorn et al., 2014). This mechanism offers a variety of possibilities to re-write how genes are traditionally expressed and creates the potential for using transcription factors and other enzymes to alter the regulation of epigenetic marks and provides the opportunity to potentially correct epigenetic disorders (reviewed in Mei et al., 2016).

### Base Editing

Base editing was the first breakthrough in the gene editing field after CRISPR/Cas9 due to the ability to perform precise point-mutation without a DSB. The first generation of base editor (BE) was BE1, a CRISPR/dCas9 fused at the *N*-terminus with a cytidine deaminase (rat APOBEC1) that produced a direct conversion of cytidine to uridine, thus effecting a C → T or G → A substitution (Komor et al., 2016). The BE1 targets deamination of nucleotides positioned within 4–8 bp that includes the PAM. However, initially BE1 was not highly effective in transitioning the U.G pair to a T.G pair due to the intermediate U.G cell repair mechanism. Dr. Liu and colleagues developed a novel BE2, a uracil DNA glycosylase inhibitor (UGI), a small protein from bacteriophage primer binding site (PBS), fused to the *C*-terminus of BE1 (Rees and Liu, 2018) to increase the efficiency of this transition. The BE2 conversion rate is three-fold higher compared to BE1 in human cells (U2OS and human embryonic kidney (HEK293T) cells), with indels formations below 0.1%. To further improve BE efficiency, the catalytic histidine residue at position 840 was restored in the Cas9 HNH domain of the BE2, creating the third-generation BE (BE3). BE3 is significantly more effective, achieving up to 37% of *C*-to-*T* conversion of total DNA (Komor et al., 2016). Since BE3, many other variants of cytidine BE have been generated resulting in improved *C*-to-*T* editing (Nishida et al., 2016; Kim et al., 2017; Komor et al., 2017; Koblan et al., 2018), including the newest BE4max and AncBE4max (up to 90% base editing efficiency) in HEK293T cells, and YFE-BE4max (up to 98%) (Koblan et al., 2018; Liu et al., 2020). These optimized BEs have been efficiently applied in mouse, rabbit, and pig embryos as well as mouse, rabbit, pig, and human cells (Kim et al., 2017; Zafra et al., 2018; Xie et al., 2019; Liu et al., 2020).

In human cells, spontaneous hydrolytic deamination of cytosine and 5-methylcytosine occurs about 100 to 500 times per day and results in the formation of uracil and thymine, respectively. This alteration may result in a permanent C.G to T.A mutations, which is known to affect about half of all pathogenic single nucleotide polymorphisms (SNPs). Adenosine base editor (ABE) is the new generation of base editor approaches that converts A.T bp to G.C bp, and has potential to revert pathogenic SNPs (Gaudelli et al., 2017). This ABE system uses laboratory-developed TadA tRNA deoxyadenosine deaminases fused with dCas9 to convert adenines into inosines. Ultimately, inosine is interpreted by polymerases as guanine (Anzalone et al., 2020). The first engineered ABE 7.8/9/10 exhibited a modest editing efficiency ranging from 1.7 to 20% in U2OS and HEK293T cells (Gaudelli et al., 2017). Genetically improved versions are able to increase the editing efficiency in HEK293T cells up to 52% using ABEmax (Koblan et al., 2018) and 69% using PAM-expanded *Sp*Cas9 variant (xCas9)-ABE7.10, and also increase the editing scope of this tool (Hu et al., 2018). Additionally, a modified ABE (ABE8e) showed the highest editing efficiency (up to 86%) in HEK293T cells (Richter et al., 2020). This technology has been applied for efficient generation of mouse model of human disease (Liu et al., 2018) and has potential to develop large animal models.

Interestingly, some studies indicated an unexpected C-to-G edits using ABE at the position 5, 6, and 7 of the protospacer (numbering beginning from the most distal position to the PAM) (Grünewald et al., 2019; Kim et al., 2019). This finding led to a new BE platform, a C-to-G base editor (CGBE1) (Kurt et al., 2020). This is the first known BE capable of introducing a transversion mutation (C→G) without a DSB. The CGBE1 was engineered from BE4max and consisted of an RNA-guided Cas9 nickase, an *E. coli*-derived uracil DNA *N*-glycosylase (eUNG) and a rat APOBEC1 cytidine deaminase variant (R33A). In HEK293T cells, highly efficient C-to-G mutation was observed with an editing frequency ranging from 41.7 to 71.5%. Moreover, they reported that C-to-G edits are more efficiently introduced in AT-rich sequences in human cells (Kurt et al., 2020). Therefore, although some of these BEs need to be improved, they may provide a powerful tool for safe gene editing *in vivo* applications to revert inherited genetic mutations.

### Point Mutation Introduction

Here, we defined point-mutation introduction as an intentional modification of target sequence with a very specific programmed mutation using either single-stranded oligodeoxynucleotide (ssODN) or double stranded donor DNA (dsDNA) to insert, delete or replace nucleotides in the target site. Targeted gene point-mutation can be genetically engineered to subvert the HDR system to introduce desired novel and controlled nucleotide modifications (deletion, insertion, or replacement of known single nucleotide or small sequences) using a customized template DNA with homologous arms (HA) to the target site (Maruyama et al., 2015; Riordan et al., 2015). With the high capability of CRISPR/Cas9 to produce DSB, both small and long template DNA can be transfected along with the CRISPR complex to promote the cell to repair the DSB by HDR using the introduced DNA template. ssODN or donor vector plasmid containing target modifications have been commonly used to perform precise alterations in many cell types (Yoshimi et al., 2016; Okamoto et al., 2019). The ssODN is a short single-strand DNA fragments containing the mutation of interest surrounded by 30 to 60 nt long homologous arms. The ssODN contains a homology sequence flanking the DSB of the targeted gene, thus, the gene is altered by knocking-in (KI) the designed mutations in the break. This approach has been successful in inserting/deleting or replacing short nucleotides (<50 bp) within the DSB (Paix et al., 2017). In mammalian cells, ssODN-mediated KIs are more effective to introduce targeted mutation than the donor plasmid approach (Yoshimi et al., 2016).

### Cas9 Tethering ssODN

Recently, Aird et al. (2018) developed a Cas9 platform to allow ssODN to be present at the moment when the CRISPR/Cas9 breaks the target sequence. This new modified Cas9 contains a fused nuclease that is a member of the endonuclease superfamily, HUH endonuclease (histidine-U-histidine with the “U” a hydrophobic residue). These endonucleases process ssDNA through a specific reaction mechanism for cleavage and ligation of recognized ssDNA site (Chandler et al., 2013; Nelson et al., 2019). These proteins contain small domains with the ability to form a covalent ligation to ssDNA. While the mechanism of this sequence binding and specificity is poorly understood, it is generally believed that it involves an identification of a DNA hairpin. The covalent bond reaction occurs at room temperature and the phosphotyrosine bond is initiated with the hydroxyl group in the tyrosine amino acid attacking the phosphate group in the ssDNA that forces the release of the nucleotides at 5′ end (Lovendahl, 2018).

Viral HUH-tags endonuclease reacts quickly with ssDNA and requires no chemical modification in their ssDNA (Lovendahl, 2018; Nelson et al., 2019). A specific HUH domain is found in the porcine circovirus 2 rep protein (PCV), a virus known to infect domestic pigs with a plasmid that originated from *Pseudomonas aeuruginosa* (Lovendahl, 2018). Aird et al. (2018) created a PCV-Cas9 that can fuse HUH-domain of PCV to either side of the Cas9 termini. Then, ssODN is designed to contain 13 nucleotides of recognition sequence at 5′ terminus to be covalently bond to PCV domain. The combination has been shown to improve the HDR up to 30-fold in both HEK293T and U2-OS cell lines targeting different genomic sequences. Nonetheless, these researchers found that a PCV fused at the *N*-terminus in the Cas9 (PCV-Cas9), resulted in a much higher point mutation efficiency than the domain fused to the *C*-terminus (Cas9-PCV). The mechanism of such difference is not fully understood. Moreover, they found that lower concentrations of Cas9-PCV RNP (1.5 pmol) enhanced the HDR efficiency up to 15- to 30-fold (Aird et al., 2018). Thus, the tethering between Cas9 to ssODN may significantly improve CRISPR/Cas9 gene editing efficiency.

### Prime Editor

The newest gene editing tool known as prime editor (PE), is one of the most accurate approaches for point-mutation introduction with great therapeutic potential to restore human genetic inherited mutations (Anzalone et al., 2019). This new concept of prime editing has been designed to insert point mutations without using a donor DNA template for the HDR pathway, or even performing a DSB in the target sequence. This gene editing tool – PE, is a catalytically impaired nCas9 (H840A) that is fused with a reverse transcriptase (RT-nCas9) with the capacity to be transfected along with a prime editing guide RNA (pegRNA). The molecular mechanism of prime editing involves the regular identification of DNA target with 20 nucleotides at the 5′ end of the pegRNA and a long 3′ end extending to interact with the opposite strand of the target sequence. The RT-nCas9 breaks the single-strand DNA via the RuvC nuclease domain. Then, the tip of the 3′ end of pegRNA, which contains a PBS, aligns against the broken DNA strand. The RT-nCas9 uses the pegRNA template containing the modification site upstream to the PBS to synthesize a brand-new sequence (Anzalone et al., 2019). Dr. David Liu’s laboratory has undoubtedly demonstrated the effectiveness of the prime editing for the introduction of targeted insertions and deletions without performing a DSB in cells (Anzalone et al., 2019). They performed 175 edits in human HEK293T cells typically achieving 20 to 50% editing efficiency, with less than 10% of indels. Prime editing holds remarkable promise for gene editing, but this technology is still immature and additional studies are needed to fully realize the prime editing potential (Yan et al., 2020).

The prime editing has been applied in mouse cells (mouse neuro-2a (N2a) cells) of which the prime editor 3 (PE3) mediated base transversion at three target sites of *Hoxd13* and androgen receptor genes with an efficiency from 8 to 40% (Liu et al., 2020). Moreover, zygote microinjection of pegRNAs, targeting the same *Hoxd13* gene led to successful conversion mutations. G-to-C and G-to-T conversions were found in 8 out of 18 (44%) and 12 out of 16 (75%) blastocysts, respectively, with mutation frequencies ranging from 1.1 to 18.5% in each embryo. Additionally, injected mouse embryos were transferred into surrogate mothers. Eight out of 30 mice contained the conversion mutation (editing efficiency of G-to-C above 1%) as well as two out of 19 mice presented conversion mutation (editing efficiency of G-to-T above 1%) (Liu et al., 2020).

### Chemically Modified ssODN

Due to the low rate of homologous recombination in the cell, different approaches were developed to improve the point-mutation efficiency through HDR pathway. Although, chemical reagents have been vastly applied (Maruyama et al., 2015; Robert et al., 2015; Vartak and Raghavan, 2015; Yu et al., 2015; Song et al., 2016; Khan et al., 2018; Kostyushev et al., 2019) to improve KI by either stimulating HDR pathway (e.g., RS-1, L755507, and Brefeldin A) or inhibiting the NHEJ (e.g., SCR7, NU7441, NU7026, KU-0060648, and VX-984), the potential adverse effects caused by these small molecules remains unknown (Okamoto et al., 2019).

Chemically modified donor oligonucleotides have also been developed to increase the KI efficiency. The ssODN has been developed by using the designed donor DNA with chemical modification of its structure. Although the mechanism by which ssODN-mediated DNA repair occurs is still not fully understood, these molecules are very useful tools for precise gene editing (Davis and Maizels, 2016; Kan et al., 2017). Renaud et al. (2016) demonstrated that subtle modifications in the ssODN can not only significantly improve gene editing efficiency but also increase the flexibility of the DNA to insert longer DNA sequences. This approach consists of the replacement of some phosphates in the ssODN sequence structure by phosphorothioate. In this molecule, one of the oxygens not involved in the phosphodiester ligation between two nucleotides is changed to Sulfur atom (S), thus, forming the phosphorothioate (O_3_PS^–3^) bond. Two of these modified phosphates are added in both 5′ and 3′ ends of the ssODN sequence. Renaud et al. (2016) reported that KI using phosphorothioate ssODN may improve gene editing efficiency up to three-fold in cell lines when compared to the conventional phosphodiester ssODN. In another study using phosphorothioate ssODN, Harmsen et al. (2018) investigated the effects of phosphorothioate in sense and antisense ssODN, as well as the presence of a single phosphorothioate in either 5′ or 3′ ends. They evaluated the efficiency of introducing a point-mutation of a single nucleotide replacement located 42 nt away from the DSB site using a 120 nt ssODN. The findings indicate that the 3′ phosphorothioate enhances gene editing by promoting integration of nucleotides away from the DSB. Also, they propose a critical role of the mismatch repair pathway at the 3′ end of ssODN that enables gene editing far away from the break, which removes the mismatch, and ssODN sequence is copied into genome (Harmsen et al., 2018).

In addition to use in ssODN, gRNAs have been adapted to be chemically synthesized as 2′-*O*-methyl-3′-phosphorothioate-modified gRNAs. The phosphorothioate results in an increase in stability and protects against exonucleases, as well as it improves gene editing efficiency of CRISPR/Cas9 to over 90% (Hendel et al., 2015; Hoellerbauer et al., 2020). Moreover, phosphorothioate-modified gRNAs have reduced off-target risk compared to the gRNA from plasmid or viral delivery (Cameron et al., 2017). The chemically modified oligonucleotide concept also led to the development of chemically modified dsDNA, which has recently been applied in HEK293T cells and led to up to 65% targeted-insertion efficiency of long fragments of DNA, discussed in the next section (Yu et al., 2020).

### Targeted Integration of Long dsDNA

Transfection or injection of long DNA fragments containing a gene of interest has been used as a strategy to express foreign genes in cells *in vitro* (Kohn et al., 1987; Bayna and Rosen, 1990) and for the production of GE animals. However, targeted integration has been a challenge due to the low rate of HDR in the cells and the high probability of random integration (reviewed by Bischoff et al., 2020). Different approaches to improve the integration of long fragments of DNA have been developed, including CRISPR/Cas9 mediating homologous recombination (HR), microhomology-mediated end-joining (MMEJ) targeted integration, homology-mediated end joining (HMEJ)-based targeted integration, and the NHEJ-mediated KI named homology-independent targeted integration (HITI) (Suzuki et al., 2016; Wu et al., 2016; Yao et al., 2017a,b). Often these approaches aim to accomplish specific targeted integration of genes of interest into what is known as safe harbor’ genes, such as Rosa26, adeno-associated virus integration site 1 (AAVS1), and H11 (Ruan et al., 2015; Wu et al., 2016; Xie et al., 2017). These sites in the genome are able to accommodate the transgene integration that ensures its high transcriptional activity in embryonic and adult tissues, and does not suppress critical endogenous genes (Ruan et al., 2015; Oceguera-Yanez et al., 2016; Weber et al., 2016; Wu et al., 2016; Yu et al., 2019; Kelly et al., 2020).

The HR was the first strategy used for targeted integration, and its approach consists of using long homologous sequences copied from the target site to induce DNA repair through the HDR pathway using the DNA template (Capecchi, 1989). The HR allows a precise mechanism for modifications of the genome of cells *in vitro* and has been extensively used to investigate gene function and to generate mouse models of human diseases (Zwaka and Thomson, 2009). The initial applications aimed to either alter the genes’ reading frame, producing gene KO, or introduce exogenous genes (KI) (Rosenthal and Brown, 2007). The ability to generate mice with specific genetic alterations has revolutionized biomedical research (Zwaka and Thomson, 2009). These targeting vectors are commonly constructed using backbone vector, such as MultiSite Gateway^®^ technology. The constructed vector contains the following basic components: either a gene of interest downstream to a constitutive promoter (e.g., cytomegalovirus promoter) or a modified target sequence; a selectable marker, which frequently is an antibiotic resistance gene (e.g., hygromycin and puromycin) or some fluorescence protein (e.g., GFP) for identification of the colonies containing the insert; the last components are homologous sequences (>500 bp each) flanking the insert (Conlon, 2006; Iiizumi et al., 2006). Once assembled, the vector is linearized for transfection into the cells using some transfection-based methods – viral particles, electroporation, lipid-mediated transfection, etc. (Kim and Eberwine, 2010). CRISPR/Cas9 co-transfected with a targeting vector could facilitate HDR by creating the DSB in the target site (Meyer et al., 2010; Sommer et al., 2014; Wu et al., 2016).

Although NHEJ and HDR are well known DNA repair pathways, a third not so popular pathway was discovered over the last decade, MMEJ pathway. MMEJ forms an alternative end-joining to repair DSB via microhomology (5 to 25 bp) between the sequences. This pathway is known to be associated with abnormalities in the cell, such as deletions, translocations, inversions, and other complex rearrangements (McVey and Lee, 2008; Yao et al., 2017b). The MMEJ pathway shares aspects with NHEJ and HDR since it joins the DSB ends without a template, like NHEJ, and MMEJ requires initial DSB end resection, similar to HDR. MMEJ initiation requires short-sequence resection of DSB ends to disclose the homologies, which also initiates HDR (Yeh et al., 2019). Moreover, MMEJ pathway seems to compete with HDR in the DNA repair, as MMEJ is active in the S and early M phases, whereas HDR is activated in late S- to G2 phase (Zhao et al., 2017; O’Brien et al., 2019). MMEJ-mediated targeted integration is also known as PITCh (Precise Integration into Target Chromosome) system (Sakuma et al., 2016) that has been shown to have an increased efficiency for targeted integration. The first studies to successfully introduce a donor plasmid by microhomology PITCh system was mediated by TALENs and CRISPR/Cas9 in silkworms and frogs (Nakade et al., 2014). In another study, PITCh system was used along with CRISPR/Cas9 for a gene cassette KI in human cells and mouse zygotes (Aida et al., 2016). They successfully knocked-in 5 kb gene cassette by MMEJ-based target integration in mice with 10% efficiency. Additionally, co-delivery of the PITCh system with *Exo1* improved KI efficiency in this study to 30%. Yao et al. (2017b) reported that MMEJ-mediated targeted integration has increased KI efficiency up to 10-fold when compared to the standard HR approach in mouse tissue. Thus, MMEJ-mediated integration is a robust approach to KI gene of interest through both *ex vivo* and *in vivo* and may offer broader applications in gene therapy (Yao et al., 2017b).

The CRISPR/Cas9-mediated HMEJ is the third alternative method for insertion of long DNA fragments into a host genome. HMEJ relies on CRISPR/Cas9-mediated cleavage of both constructed transgene vector and target genome site. The donor plasmid contains HAs with approximately 800 bp and the targeted genome gRNA site at the 5′ end of the left HA, as well as the 3′ end of the right HA (Banan, 2020). This strategy may take advantage of HDR pathway as well as a HMEJ pathway (Yao et al., 2017a). Yao et al. (2017a) demonstrates that HMEJ strategy provides the highest targeted integration efficiency (up to 27% KI) when compared to HR, MMEJ, and NHEJ approaches in HEK293T cells, mouse primary astrocytes, and neurons cells, as well as mouse and monkey embryos.

The newest potential approach for targeted integration is the HITI. This method is a NHEJ-mediated KI, which works independent from HDR for targeted insertion and provides a robust donor vector for both dividing and non-dividing cells (Suzuki et al., 2016). This concept has been highly efficient to KI donor vectors with low rates of off-target mutations *in vitro* and *in vivo* (Suzuki and Belmonte, 2018). The method is based on the transfection of a minicircle vector produced from pre-minicircle plasmids containing the target site of CRISPR/Cas9 inside of the minicircle. Suzuki et al. (2016), demonstrated the potential of HITI with 56% efficiency of targeted insertion of IRESmCherry in mouse neurons, while keeping the indels mutations at the same target site at the low level (5 to 10%). Moreover, their findings present high on-target specificity of HITI (90–95%). Among all evaluated cells, 30–50% showed biallelic transgene integration (Suzuki et al., 2016). Shi et al. (2020) applied HITI along with CRISPR/Cas9 targeting to the ovalbumin (OVA) locus in chicken DF-1 and embryonic fibroblast cells. EGFP cassette was introduced into the OVA locus via HITI and the GFP expression activated by endogenous OVA promoter using the dCas9-VPR transactivating approach (Shi et al., 2020). In another study, an efficient transgenesis using HITI was performed in ferret embryos. An 8 kb cassette expressing Tomato/EGFP was inserted into intron 1 of the Rosa26 locus. Zygotes (*n* = 151) were microinjected with the plasmid and CRISPR/Cas9 RNP. Five out of 23 offspring exhibited the reporter expression (Yu et al., 2019). Therefore, HITI method offers a great enhancement over the other methods as it takes advantage of NHEJ for gene insertion.

Gene insertion approaches have received a new endorsement using chemically modified oligonucleotides. Recently, Yu et al. (2020) inserted different types of modifications into dsDNA to evaluate the effect of chemically modified dsDNA to improve gene insertion into target integration site. The recent results demonstrate that using short homologous arms (50 bp) containing 5′-modified double-stranded modification, the KI rates for long inserts (2.5 kb) was up to 40%, whereas for short inserts (0.7 kb) reached an unprecedented rate of 65% in HEK293T cells. Moreover, up to five-fold increase of gene KIs was observed in different loci of human cancer and stem cell genomes. The chemical modification that provided such an improvement was a C6-PEG10 at the 5′ end of each homologous arm (Yu et al., 2020). Although the approach has not been tested in other cell types, including animals, the chemically modified dsDNA may become a solution for insertion of gene of interest in the target sequence with higher efficiency when compared to the traditional approaches.

## Production of Gene Edited Farm Animals

### Zygote Manipulation

The first Genetically Engineered (GE) farm animals were produced 35 years ago by DNA microinjection into the pronucleus of zygotes (Hammer et al., 1985). Transgenic animals were successfully produced in several species including mice (Gordon et al., 1980), rabbits, pigs, sheep, cattle, and goats by injection of genes of interest into the pronucleus of a zygote (review by Wall, 1996). At that time, this technique was suffering from several serious limitations (Wilmut and Clark, 1991; Pursel and Rexroad, 1993). The most profound constraint was that DNA can only be added, not deleted, or modified *in situ*. Also, the integration of foreign DNA was random leading to erratic transgene expression due to the integration site effect. Furthermore, random integration has a risk for the disruption of essential endogenous DNA sequences or activation of cellular oncogenes, both of which could have deleterious effects on the animal’s health. Finally, GE animals generated using zygote microinjection are commonly mosaic, i.e., when desired genetic alteration is not present in all cells (Wieland et al., 1990). Therefore, the production of the required phenotype coupled to germ line transmission could require the generation of several transgenic founder lines followed by breeding.

Advances in CRISPR/Cas9 genome editing significantly improved the ability to precisely disrupt genes and/or introduce specific mutations by direct zygote manipulation (pronuclear or cytoplasmic injection, or electroporation; Navarro-Serna et al., 2020). Recently, a high efficiency of generating indels mutations in bovine and porcine zygotes via electroporation was reported (Miao et al., 2019). This method greatly simplifies generation of GE livestock as it does not require micromanipulation expertise. However, genetic mosaicism continues to be a major challenge using zygote manipulation approach (reviewed by Mehravar et al., 2019). Mosaicism emerges when DNA replication precedes CRISPR-mediated genome edition, which greatly reduces the likelihoods for direct KO generation. The impact of mosaicism could be even more devastating if both somatic and germline mosaicism are present in the offspring. One of the approaches proposed to reduce genetic mosaicism is an introduction of CRISPR/Cas9 into either metaphase II (MII) oocyte or a very early zygote stage. Electroporation of Cas9 RNP into an early zygote stage has eliminated mosaic mutants in mice (Kim et al., 2014; Hashimoto et al., 2016). However, injection of CRISPR/Cas9 into MII oocytes did not reduce mosaicism compared to the zygote injection in sheep and cattle (Lamas-Toranzo et al., 2019; O’Neil et al., 2020). Inability of CRISPR to recognize its target locus prior to some degree of chromatin de-condensation took place might be a reason for these somewhat surprising outcomes.

Shortening longevity of Cas9 by accelerating its degradation is another possible tactic for reducing mosaicism. This can be accomplished by tagging Cas9 with ubiquitin-proteasomal degradation signals that facilitate the Cas9 degradation. Alternatively, to completely eliminate the risk of mosaicism nuclear transfer approach using GE cells could be considered.

### Somatic Cell Nuclear Transfer – Cloning

Somatic cell nuclear transfer was initially developed in sheep with the birth of Dolly in 1996 (Wilmut et al., 1997). The technology was later established for other key livestock species: cattle (Cibelli et al., 1998), goats (Baguisi et al., 1999), pigs (Polejaeva et al., 2000), and equine (Woods et al., 2003), providing the first cell-mediated platform for livestock genetic engineering. Precise genetic manipulations are introduced in somatic cells (typically fetal fibroblasts), followed by the isolation of single-cell-derived colonies and cell screening to confirm that the desired genetic modifications are present in the cells. Subsequently, the cells are used as donor cells for SCNT (Schnieke et al., 1997; Clark et al., 2000; McCreath et al., 2000; Dai et al., 2002; Phelps et al., 2003). This method has a major advantage compared with zygote manipulation approach for GE animal production, because the entire animal is derived from a single GE donor nucleus, thus the risk of mosaicism is eliminated (Polejaeva and Campbell, 2000). However, this method is more technically challenging and typically has a low term development rate. Additionally, potential cloning related epigenetic alterations might contribute to the GE animal phenotype, thus generation of F1 animals is often desirable for a proper characterization of GE models. Despite these limitations, SCNT continues to be the primary method for the production of the KI gene edited livestock, with nearly 70% of the published work was conducted using this methodology (Table 3). Additionally, about half of the published KO farm animals were generated using SCNT (Tables 1, 2). GE animals produced by SCNT often required the use of fewer recipient animals compared to the number of animals needed for the zygote micromanipulations (Schnieke et al., 1997).

**TABLE 1:** CRISPR-meditated gene knockout in livestock: agricultural applications.

Species	*Gene*	Purpose of manipulation	Approach	Mosaicism (%)	References
Sheep	*ASIP*	Coat color pattern	MI	2/5 (40.0%)	Zhang X. et al. (2017)
	*FGF5*	Wool growth	MI	(6.3–100%)	Hu et al. (2017), Li W. R. et al. (2017), Zhang R. et al. (2020)
	*MSTN, ASIP, and BCO2*	Economically important traits	MI	2/2 (100%)	Wang X. et al. (2016b)
	*MSTN*	Meat production	MI or SCNT	(0–100%)	Deng et al. (2014); Crispo et al. (2015), Zhang Y. et al. (2019); Yi et al. (2020)
Goat	*BLG*	Milk quality	MI	3/4 (75.0%)	Zhou et al. (2017)
	*MSTN and FGF5*	Meat and cashmere production	MI	5/10 (50.0%)	Wang X. et al. (2015a)
	*MSTN*	Meat production	MI or SCNT	(0–100%)	Ni et al. (2014); Guo et al. (2016), He et al. (2018); Zhang Y. et al. (2019)
	*NANOS2*	Surrogate sires for genetic dissemination	SCNT	N/A	Ciccarelli et al. (2020)
	*EDAR*	Cashmere yield	SCNT	N/A	Hao et al. (2018)
Pig	*IGF2 regulatory element*	Meat production	MI (nCas9)	6/6 (100%)	Xiang et al. (2018)
	*NANOS2*	Surrogate sires for genetic dissemination	MI	6/18 (33.3%)	Park et al. (2017)
	*ANPEP*	Viral resistance	MI	1/9 (11.1%)	Whitworth et al. (2019)
	*CD163*	Resistance to PRRS virus	MI, EP, or SCNT	No	Whitworth et al. (2014); Yang et al. (2018), Tanihara et al. (2019)
	*IRX3*	Reduced fat content in Bama minipigs	SCNT	N/A	Zhu et al. (2020)
	*NANOS2*	Surrogate sires for genetic dissemination	SCNT	N/A	Ciccarelli et al. (2020)
	*MSTN*	Meat production	SCNT	N/A	Wang K. et al. (2015), Wang K. et al. (2017), Li R. et al. (2020)
	*CD163 and pAPN*	Viral resistance	SCNT	N/A	Xu et al. (2020)
	*FBXO40*	Meat production	SCNT	N/A	Zou et al. (2018)
Cattle	*NANOS2*	Surrogate sires for genetic dissemination	MI	1/3 (33.3%)	Ciccarelli et al. (2020)

*SCNT, somatic cell nuclear transfer; MI, zygote microinjection; EP, zygote electroporation; nCas9, Cas9 nickase; N/A, not applicable.*

**TABLE 2:** CRISPR-meditated gene knockout in livestock: biomedical applications.

Species	*Gene*	Purpose of manipulation	Approach	Mosaicism (%)	References
Sheep	*PDX1*	Pancreas-deficient model development	MI	2/2 (100%)	Vilarino et al. (2017)
	*BCO2*	b-carotene metabolism research	MI	2/6 (33.3%)	Niu Y. et al. (2017)
	*CFTR*	Cystic fibrosis model	SCNT	N/A	Fan et al. (2018a)
Goat	*IGHM*	Human polyclonal antibody production	SCNT	N/A	Fan et al. (2018b)
Cattle	*GGTA and CMAH*	Xenotransplantation	SCNT	N/A	Perota et al. (2019)
Pig	*SCD5*	Chronic Maxillary Sinusitis and Dysostosis diseases	MI	No	Carey et al. (2019)
	*CMAH*	Viral resistance	MI	3/5 (60.0%)	Tu et al. (2019)
	*Ig-J_*H*_*	Hepatitis E virus pathogenicity	MI	No	Yugo et al. (2018)
	*ULBP1*	Xenotransplantation	MI (nCas9)	No	Joanna et al. (2018)
	*TMPRSS2*	Resistance to influenza viruses	MI	5/12 (41.7%)	Whitworth et al. (2017)
	*PDX1*	Lack of pancreas, regenerative medicine	MI	2/3 (66.6%)	Wu et al. (2017)
	*DMD*	Duchenne muscular dystrophy model	MI	1/1 (100%)	Yu et al. (2016)
	*PARK2, DJ-1, and PINK1*	Parkinson’s disease model	MI	2/2 (100%)	Wang X. et al. (2016a)
	*RAG2 and IL2RG*	Model for severe combined immunodeficiency	MI	3/17 (17.6%)	Lei et al. (2016)
	*NPC1L1*	Human cardiovascular and metabolic diseases	MI	5/11 (45.5%)	Wang Y. et al. (2015)
	*MITF*	Human Waardenburg and Tietz syndromes	MI	No	Wang X. et al. (2015b), Hai et al. (2017)
	*vWF*	Model of von Willebrand disease	MI	Most pigs	Hai et al. (2014)
	*EDA*	Lung disease model	MI	No	Ostedgaard et al. (2020)
	*GRB10*	GRB10 role in insulin resistance and obesity	MI or EP	No	Sheets et al. (2016)
	*GGTA1*	Xenotransplantation	MI or EP	0–40.0%	Petersen et al. (2016); Chuang et al. (2017), Tanihara et al. (2020)
	*TP53*	Model with tumor phenotypes	EP	5/6 (83.3%)	Tanihara et al. (2018)
	*IL2RG*	Immunodeficiency model	SCNT	N/A	Ren et al. (2020)
	*SIX1 and SIX4*	Kidney-deficient model	SCNT	N/A	Wang J. et al. (2019)
	*B2M*	Xenotransplantation	SCNT	N/A	Sake et al. (2019)
	*GGTA1*,β*4GalNT2, CMAH*	A source of Bioprosthetic heart valves	SCNT	N/A	Zhang R. et al. (2018)
	*ApoE*	Models of atherosclerosis	SCNT	N/A	Fang et al. (2018)
	*INS*	Diabetes research	SCNT	N/A	Cho et al. (2018)
	*TPH2*	5-HT deficiency and behavior abnormality	SCNT	N/A	Li Z. et al. (2017)
	*Hoxc13*	Ectodermal dysplasia–9 disease	SCNT	N/A	Han et al. (2017)
	*GGTA1 and CMAH*	Xenotransplantation	SCNT or sSCNT	N/A	Fischer et al. (2016); Gao et al. (2016)
	*PERV*	PERV-inactivated animals, xenotransplantation	SCNT	N/A	Niu D. et al. (2017)
	*C3*	Roles of C3 in human diseases	SCNT	N/A	Zhang W. et al. (2017)
	*IL2RG*	Severe combined immunodeficiency	SCNT	N/A	Kang et al. (2016a)
	*RUNX3*	Cancer model	SCNT	N/A	Kang et al. (2016b)
	*Ig-J_*H*_*	B cell-deficient model for h Ab production	SCNT	N/A	Chen et al. (2015)
	*TYR*	Oculocutaneous albinism type 1 disease	SCNT (nCas9)	N/A	Zhou et al. (2015)
	*PARK2 and PINK1*	Parkinson’s disease	SCNT	N/A	Zhou et al. (2015)
	*GGTA1, CMAH & iGb3S*	Xenotransplantation	SCNT	N/A	Li et al. (2015)
	*CD1D*	Models for biomedicine	SCNT	N/A	Whitworth et al. (2014)
	*Class I MHC*	Model for immunological research	SCNT	N/A	Reyes et al. (2014)
	*ApoE and LDLR*	Human cardiovascular disease	SCNT	N/A	Huang et al. (2017)

*SCNT, somatic cell nuclear transfer; sSCNT, serial SCNT; MI, zygote microinjection; EP, zygote electroporation; nCas9, Cas9 nickase; N/A, not applicable.*

**TABLE 3:** CRISPR-mediated gene knockin in livestock.

Species	*Gene*	Purpose of manipulation	Type of KI	Approach	SCNT or MI	KI Animals produced	Mosaicism (%)	References
		Agriculture: improvements in						
Sheep	*SOCS2*	Reproductive traits	Point mutation	Crispr/Cas9 BE	MI	3/4 (25%)	3/3 (100%)	Zhou et al. (2019)
	*BMPR1B*	Reproductive traits	Point mutation	Crispr/Cas9	MI	5/21 (23.8%)	Not stated	Zhou et al. (2018)
Goat	*T*β*4*	CCR5-targeted KI, cashmere yield	Gene insertion	Crispr/Cas9	SCNT	1	N/A	Li X. et al. (2019)
	*FGF5*	Cashmere yield	Point mutation	Crispr/Cas9 BE	MI	5/5 (100%)	5/5 (100%	Li G. et al. (2019)
	*GDF9*	Reproductive traits	Point mutation	Crispr/Cas9	MI	4/17 (23.5%)	2/4 (50.0%)	Niu et al. (2018)
	*FAT-1*	Disease resistance	Gene insertion	Crispr/Cas9	SCNT	1 from 8 pregnancies	N/A	Zhang J. et al. (2018)
Cattle	*Pc*	Generation of a polled genotype	Gene insertion	Crispr/Cas12a	SCNT	1, died on D1 after birth	N/A	Schuster et al. (2020)
	*NRAMP1*	Tuberculosis resistance	Gene insertion	Crispr/Cas9n	SCNT	9	N/A	Gao et al. (2017)
	*IARS*	Correction of IARS syndrome	Gene insertion	Crispr/Cas9	SCNT	5 viable fetuses	N/A	Ikeda et al. (2017)
Pig	*PBD-2*	Disease-resistant pigs	Gene insertion	Crispr/Cas9	SCNT	5 pigs	N/A	Huang et al. (2020)
	*MSTN*	Meat production	Gene insertion	Crispr/Cas9	SCNT	2 pigs	N/A	Zou Y.-L. et al. (2019)
	*UCP1*	Reproduction traits	Gene insertion	Crispr/Cas9	SCNT	12 piglets	N/A	Zheng et al. (2017)
	*MSTN*	Meat production	Point mutation	Crispr/Cas9	SCNT	1 stillborn piglet	N/A	Wang K. et al. (2016)
	*MSTN*	MSTN-KO without selectable marker	Gene insertion	Crispr/Cas9	SCNT	2 piglets	No	Bi et al. (2016)
	*RSAD2*	Generation of pigs with viral resistance	Gene insertion	Crispr/Cas9	SCNT	1 pig	No	Xie et al. (2020)
		Biomedical applications:						
Sheep	*ALPL*	Model of hypophosphatasia	Point mutation	Crispr/Cas9	MI	6/9 (66.6%)	No	Williams et al. (2018)
	*PPT1*	Infantile neuronal ceroid lipofuscinoses	Point mutation	Crispr/Cas9	MI	6/24 (25.0%)	Not stated	Eaton et al. (2019)
	*tGFP*	Rosa26-targeted KI	Gene insertion	Crispr/Cas9	MI	1/8 (12.5%)	Not stated	Wu et al. (2016)
	*OTOF*	Hearing loss phenotype	Point mutation	Crispr/Cas9	MI	8/73 (11.0%)	2/8 (25.0%)	Menchaca et al. (2020b)
Cattle	*CMAH*	Xenotransplantation	Point mutation	Crispr/Cas12a	SCNT	2	N/A	Perota et al. (2019)
Pig	*hF9*	Gene therapy for hemophilia B pigs	Gene insertion	Crispr/Cas9	SCNT	5 pigs	N/A	Chen et al. (2020)
	*BgEgXyAp*	Salivary gland as bioreactor	Gene insertion	Crispr/Cas9	SCNT	4 piglets (1/4 alive)	N/A	Li G. et al. (2020)
	*hIAPP*	Type 2 diabetic miniature pig model	Gene insertion	Crispr/Cas9	SCNT	24	N/A	Zou X. et al. (2019)
	*SNCA*	Parkinson’s disease model	Gene insertion	Crispr/Cas9	SCNT	8 piglets	N/A	Zhu et al. (2018)
	*HTT*	Huntingtin KI model	Gene insertion	Crispr/Cas9	SCNT	6 piglets	N/A	Yan et al. (2018)
	*GGTA1*	Xenotransplantation	Gene insertion	*Fok*I-dCas9	SCNT	2 piglets	N/A	Nottle et al. (2017)
	*tdTomato*	porcine Oct4 reporter system	Gene insertion	Crispr/Cas9	SCNT	2 piglets	N/A	Lai et al. (2016)
	*hALB*	Tg animals as bioreactors	Gene insertion	Crispr/Cas9	MI	16/16 (100%)	1/16 (6.3%)	Peng et al. (2015)
	*GFP*	H11-targeted KI	Gene insertion	Crispr/Cas9	SCNT	1 piglet	N/A	Ruan et al. (2015)

*SCNT, somatic cell nuclear transfer; MI, zygote microinjection; BE, base editing; N/A, not applicable.*

## Gene Editing Applications in Agriculture

The global demand for animal products is substantially growing, driven by a combination of burgeoning population, urbanization, and income growth. However, approximately one billion people in the world are still chronically malnourished (Godfray et al., 2010). Global climate change will only exacerbate the lack of animal protein production (McMichael, 2012). Present efforts to satisfy global food needs are degrading an already burdened environment (Foley et al., 2011; Tilman et al., 2011). Improvements in the efficiency of animal production and food safety are becoming more important considerations for protection of the environment and reduction in land usage (Clark and Whitelaw, 2003). The United Nations (UN) predicts world population will reach 9.8 billion by mid-century (United Nations, 2020), and therefore, calls for use of innovative strategies and new technologies to double food production by 2050 in order to meet demand from the world’s growing population. According to the UN, this increased production must come from virtually the same land area as today. Thus, the need for innovation through new technologies is essential for the future of people, communities, and natural resources. The recent development of gene editing combined with the animal production technologies provide the potential for accelerating the genetic improvement of livestock, including alteration of production traits, enhancing resistance to disease, reducing the threat of zoonotic disease transmission, and improvement of livestock welfare (Tan et al., 2013). Genetic-based increases in sustainable animal productivity will be a key to meet the global food demand.

### Improving Livestock Production Traits

**FIGURE 2:**
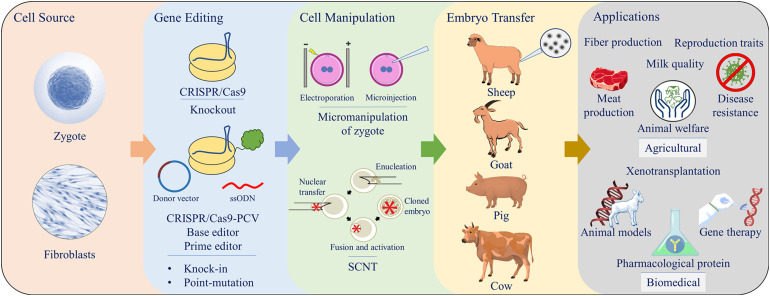
Schematic summary of CRISPR/Cas9 gene editing using either zygote micromanipulation (electroporation or microinjection) or somatic cell nuclear transfer (SCNT) for generation of livestock animals for various applications.

Examples of gene editing application for livestock production trait improvements are provided in this section. Additionally, a comprehensive summary included in Tables 1 and 2. Key interest areas covered under agricultural umbrella include meat and fiber production, improvements in milk quality, and reproductive performance, as well as disease resistance and animal welfare (Figure 2).

Myostatin (MSTN), a negative regulator of skeletal muscle mass (McPherron et al., 1997) is the most frequent target of gene editing, as *MSTN* KO offers a strategy for promoting animal muscle growth in livestock production. Myostatin (previously called GDF-8) was originally identified in a screen for new members of the TGF-ß superfamily in mammals (McPherron et al., 1997). In adult tissues, myostatin is expressed almost exclusively in skeletal muscle, but clearly detectable levels of myostatin RNA are also present in adipose tissue (Roberts and Goetz, 2003; Lee, 2004). The function of myostatin was elucidated through gene KO studies, in which myostatin KO mice have about a doubling of skeletal muscle weights throughout the body as a result of a combination of muscle fiber hyperplasia and hypertrophy (McPherron et al., 1997). The myostatin gene has been analyzed in many different species and has been found to be extraordinarily well conserved. Natural gene mutations of MSTN have also been reported in some cattle breeds (Grobet et al., 1997, 1998), sheep (Boman et al., 2009), dogs (Mosher et al., 2007), and human (Schuelke et al., 2004). These animals show a double-muscled phenotype of dramatically increased muscle mass, and still viable and fertile (Grobet et al., 1997, 1998; Mosher et al., 2007; Boman et al., 2009). Moreover, pharmacological agents capable of blocking MSTN activity have been shown to cause significant increases in muscle growth when administered systemically to adult mice (Bogdanovich et al., 2002; Whittemore et al., 2003; Lee et al., 2005), demonstrating that MSTN plays a critical role in regulating muscle homeostasis postnatally by suppressing muscle growth. Successful disruption of the MSTN gene by gene editing was reported in sheep, goats, and pigs that lead to enhance animal growth performance (Deng et al., 2014; Ni et al., 2014; Wang K. et al., 2015).

Another potential candidate gene for improving meat production in livestock and for developing therapeutic interventions for muscle diseases is *FBXO40* protein coding gene, a member of the F-box protein family. Expression of FBXO40 is restricted to muscle, and mice with an *Fbxo40* null mutation exhibit muscle hypertrophy. FBXO40 KO pigs have been recently produced but exhibited only marginal increase in muscle mass (4%) compared to WT controls (Zou et al., 2018). The KO pigs developed normally, and no pathological changes were found in major organs.

The whey protein β-lactoglobulin (BLG) is a major milk allergen which is absent in human milk. *BLG* KO goat and cows have been produced by CRISPR/Cas9 and zygote microinjection and ZFNs gene editing and SCNT, respectively (Zhou et al., 2017; Sun et al., 2018). Western blot results showed that the BLG protein had been abolished in the milk of the BLG KO goat. In comparison with WT goats, BLG KO goats have exhibited a decreased level of fat, protein, lactose, and solid not fat in the milk by 5.49, 7.68, 7.97, and 7.7%, respectively.

In several studies two or three genes were targeted simultaneously leading to double or triple gene KOs (Wang X. et al., 2016b). For instance, MSTN and FGF5 KO goats were produced to improve meat production and cashmere yield (Wang X. et al., 2015a). Fibroblast growth factor 5 (FGF5), a secreted signaling protein that inhibits hair growth by blocking dermal papilla cell activation and is regarded as the causative gene underlying the angora phenotype (long hair coat). The efficiency of disrupting MSTN and FGF5 in 98 tested animals was 15 and 21%, respectively, and 10% of the animals had double gene KOs.

A concept of “surrogate sires” was recently validated for pigs, goats, and cattle (Ciccarelli et al., 2020) by demonstrating that the *NANOS2* gene KO males generated by CRISPR/Cas9 editing have testes that are germline ablated but otherwise structurally normal. Subsequent, spermatogonial stem cell transplantation (SSCT) with allogeneic donor stem cells led to sustained donor-derived spermatogenesis. This prove of principle study has great potential for dissemination of elite livestock genetics.

### Improving Health and Welfare

Porcine reproductive and respiratory syndrome virus (PRRSV) causes severe economic losses to current swine production worldwide. Highly pathogenic PRRSV (HP-PRRSV), originated from a genotype 2 PRRSV, is more virulent than classical PRRSV and further exacerbates the economic impact. Several groups successfully generated CD163 KO pigs using CRISPR/Cas9 gene editing (Whitworth et al., 2014; Yang et al., 2018; Tanihara et al., 2019). Challenge with either the NVSL 97-7895 PRRSV virulent virus isolate (Whitworth et al., 2016) or the HP-PRRSV strain (Yang et al., 2018) showed that CD163 KO pigs are completely resistant to viral infection manifested by the absence of viremia, antibody response, high fever or any other PRRS-associated clinical signs. By comparison, wild-type (WT) controls displayed typical signs of PRRSV infection (Whitworth et al., 2016; Yang et al., 2018). More recently, Whitworth et al. showed that amino peptidase N (APN) deficient pigs are fully resistant to transmissible gastroenteritis virus (TGEV), but not porcine epidemic diarrhea virus (PEDV) (Whitworth et al., 2019). Additionally, porcine alveolar macrophages derived from the APN-deficient pigs showed resistance to porcine deltacoronavirus (PDCoV). However, lung fibroblast-like cells derived from these animals supported a high level of PDCoV infection indicating that APN is a dispensible receptor for PDCoV (Stoian et al., 2020).

Double-gene-knockout (DKO) pigs containing KOs for known receptor proteins CD163 and pAPN are reported to be completely resistant to genotype 2 PRRSV and TGEV (Xu et al., 2020). Additional infection challenge experiments have shown that these DKO pigs exhibit decreased susceptibility to PDCoV, thus providing *in vivo* evidence that pAPN as likely to be one of PDCoV receptors.

Prion diseases, such as scrapie in goats or sheep, bovine spongiform encephalopathy (BSE) in cattle and Creutzfeldt-Jakob disease (CJD) in humans, are a group of fatal and infectious neurodegenerative disorders of the central nervous system (CNS) (Prusiner, 1998). There is considerable evidence that the prion diseases are caused by propagation of misfolded forms of the normal cellular prion protein (PrP) (Aguzzi et al., 2008). The pathogenic form of this protein appears to be devoid of nucleic acids and supports its own amplification in the host. This self-propagating process allows for the exponential increase and accumulation of misfolded PrP in cells, resulting in a disruption of cell function and ultimately cell death (Aguzzi et al., 2008). Prion diseases have had important economic impact, resulting in billions of dollars in lost earnings in many countries due to trade embargos and weakened consumer confidence. This has energized efforts to understand prion diseases as well as to develop tools for disease detection, prevention, and management. More interestingly, while the cellular PrP is absolutely required for disease pathogenesis, it is dispensable for normal animal development. Disruption of PrP expression in mice resulted in no apparent developmental abnormalities (Bueler et al., 1993; Manson et al., 1994). Moreover, cattle devoid of PrP are clinically, histopathologically, immunologically, and physiologically normal, and the brain tissue homogenates from PrP KO cattle are resistant to prion propagation *in vitro* (Richt et al., 2007). PrP KO livestock will improve food safety, which will potentially relieve food crisis in the future (Ni et al., 2014).

Disease causing mutations can also be effectively corrected using gene editing techniques. Ikeda et al. were able to repair a recessive mutation responsible for isoleucyl-tRNA synthetase (IARS) syndrome in Japanese Black cattle (Ikeda et al., 2017). Selective breeding for more than 60 years has yielded high meat quality famous for its distinctive marbling but has also resulted in the accumulation of recessive mutations that cause genetic diseases. The c.235G > C (p.Val79Leu) substitution in the *IARS* gene causes a 38% reduction in the aminoacylation activity of the IARS protein, which impairs protein synthesis. Homozygous mutant calves exhibit neonatal weakness with intrauterine growth retardation.

In modern livestock, daily management of horned cattle pose a high risk of injury for each other as well as for the farmers. Dehorning is associated with stress and pain for the calves and raises concerns regarding animal welfare. Naturally occurring structural variants causing polledness are known for most beef cattle. Polled Celtic variant from the genome of an Angus cow was isolated and integrated into the genome of fibroblasts taken from the horned bull using the CRISPR/Cas12a system, followed by SCNT (Schuster et al., 2020). The study successfully demonstrated practical application of CRISPR/Cas12a in dairy husbandry.

## Biomedical Applications

GE livestock models play a critical role in advancing our understanding of disease mechanisms due to their anatomical and physiological similarity to humans, and thus, are likely to open new clinically relevant mechanism-based targets for the prevention and treatment of numerous diseases. Livestock models have undoubtedly made a significant contribution in translational medicine. They effectively represent the complexity of outbred species and often have more similar pathogenesis of genetic, metabolic, infectious, and neoplastic diseases to those in human compared with the mouse model equivalents (Roth and Tuggle, 2015; Polejaeva et al., 2016). Similar organ size and function make them more suitable than a mouse for many biomedical applications, such as tissue recovery, serial biopsies, and blood sampling, device development, whole-organ manipulations, cloning, and the development of surgical procedures (Reynolds et al., 2009). Current availability of genome sequences and efficient gene-editing techniques are increasing accessibility of GE livestock models for biomedical research, xenotransplantation, and gene therapy. Numerous review papers discussing the topic of engineering large animal models are available (Whitelaw et al., 2016; Hamernik, 2019) including reviews on gene-editing for xenotransplantation (Meier et al., 2018; Cowan et al., 2019). GE swine models have been made available to researchers through institutions such as the National Swine Resource and Research Center at the University of Missouri–Columbia (http://www.nsrrc.missouri.edu, accessed 29 September 2020) and the Meiji University International Institute for Bio-Resource Research (MUIIBR) in Japan (http://www.muiibr.com, accessed 30 September 2020). Here, we provide a list of livestock models recently generated by CRISPR/Cas9 (Tables 2, 3). The pig is increasingly gaining approval and it is the most frequently used large biomedical model (Gutierrez et al., 2015). Porcine gene-edited models represent aproximaly 80% of all GE livestock models (Tables 2, 3).

Cattle are commonly used as a model for human female reproduction, including ovarian function, the effect of aging on fertility, and embryo–maternal communication (reviewed in Polejaeva et al., 2016). Similarities between sheep and humans in the physiological parameters of lung function, such as airflow, resistance, and breathing rates, have made sheep a valuable model for asthma research (Van der Velden and Snibson, 2011). Furthermore, preterm and term lambs have similar pulmonary structure, including airway branching, submucosal glands, and a dual oxidase (Duox)–lactoperoxidase (LPO) oxidative system, as well as prenatal alveologenesis that make them an ideal model to study respiratory distress syndrome in preterm infants (Liggins and Howie, 1972) and respiratory syncytial virus (RSV) infection (Derscheid and Ackermann, 2012). Ovine model of Cystic Fibrosis (CF) could be also very valuable to study developmental progression of CF (Fan et al., 2018a). Advancement in gene editing technology will further accelerate development of new more sophisticated large animal models allowing to study different aspects of various human diseases.

## Discussion

Initial studies in livestock have primarily utilized CRISPR/Cas9 NHEJ mechanism for disruption of genes of interest (KO) via indels introduction (Tables 1, 2). More recently, farm animals with point mutations and gene insertions (KI) have been successfully produced using ssODN donor sequences, CRISPR/Cas9 base editing and CRISPR/Cas9 nickase approaches (Table 3). The applications of gene editing technologies for generation of livestock are very diverse, ranging from enhancing important production traits such as meat, milk, and fiber production (Deng et al., 2014; Crispo et al., 2015; Hu et al., 2017; Zhou et al., 2017; Zhang R. et al., 2020) to improving disease resistance, health, reproductive efficiency, facilitating animal welfare, and developing new biomedical models to better understand the etiology of diseases and develop novel mechanism-based therapeutic approaches (Vilarino et al., 2017; Fan et al., 2018a,b; Tu et al., 2019; Tanihara et al., 2020).

Newly developed gene editing tools (cytosine base editor, CBE and ABE) facilitate the generation of point-mutations without DSB. They can introduce four types of transition mutations (C → T, A → G, T → C, and G → A), which cover approximately 30% of all known human pathogenic variants (Anzalone et al., 2020), so the use of these tools could be increasingly beneficial for gene therapy. The CRISPR/Cas9 platform can also be used to modulate gene expression and impact epigenetics (Gilbert et al., 2013; Lawhorn et al., 2014). This mechanism offers a variety of possibilities to re-write how genes are traditionally expressed and provides the opportunity to use transcription factors and other enzymes in the regulation/modification of epigenetic marks and correcting epigenetic disorders (reviewed in Mei et al., 2016). Prime Editing technology has shown that all 12 combinations of base changes (transition and transversion) are possible without performing a DSB in cells (Anzalone et al., 2019). This gene editing tool is a catalytically impaired nCas9 (H840A) fused with a reverse transcriptase (RT-nCas9) that is transfected along with a pegRNA. Several strategies have been developed to improve the integration efficiency of long DNA fragments, including CRISPR/Cas9 mediating HR, MMEJ targeted integration, HMEJ targeted integration, and the NHEJ-mediated KI named HITI. HITI has the highest on-target specificity (90–95%) with biallelic integration of transgene ranging between 30 and50% *in vitro* in several cell types including dividing (HEK293) and non-dividing (mouse primary neurons) cells. Furthermore, HITI approach led to the successful DNA KI *in vivo* demonstrating the efficacy of HITI in improving visual function using a rat model of retinitis pigmentosa (Suzuki et al., 2016). The robustness of this approach is likely to be translatable to the livestock species. The use of chemically modified ssODN, such as phosphorothioate, is highly efficient method for the introduction of point mutations and/or single nucleotide replacements that could be very useful for correction of pathogenic mutations in livestock, and developing animal models of human disease or testing gene therapy strategies.

While editing scope and efficiency of CRISPR/Cas9 and its variants continue to improve, potential introduction of off-target mutations remains the major concern when producing animals for agriculture or using them in biomedical applications (Zhang X. H. et al., 2015). These off-target sites are sequences similar to the gRNA sequence except for up to four mismatched mutations that can be tolerated by CRISPR/Cas9 (Haeussler, 2020). The tolerance for mismatch pairing may cause attack by CRISPR/Cas9 during gene editing, which ultimately may lead to an introduction of unintended mutations. Off-target mutations may result in a silent mutation or produce a loss of function in coding regions. Nonetheless, the concerns are in the formation of an aberrant form of protein that induces food allergenicity or affect animal health if unintended genetic modifications could lead to tumor formation due to disruption of mechanisms such as a tumor suppressor gene (Ishii, 2017). Up to thousands off-target mutations have been found in previous studies in gene edited cells, embryos, and animals (Crispo et al., 2015; Kim et al., 2015; Tsai et al., 2015; Wang X. et al., 2015a; Carey et al., 2019; Zuo et al., 2019; Haeussler, 2020; Zuccaro et al., 2020), which raise the importance on investigating in-depth the gene editing approaches for reduction of those mutations. For instance, the use of CRISPR/Cas9 RNP instead of a plasmid vector, reduced the risk of off-target mutations as RNP is cleared from the cells within 24 hours after transfection (DeWitt et al., 2017). Furthermore, other methods are in development to minimize the off-target effects such as CRISPR Guide RNA Assisted Reduction of Damage (GUARD) that protects off-target sites by co-delivering short gRNAs directed against off-target loci by competition with the on-target gRNA without affecting on-target editing efficiency (Coelho et al., 2020). Nonetheless, it would be appropriate to investigate off-target mutations in animals, embryos or somatic cells as deeply as possible using methods for identification of off-target sites, such as whole genome sequencing (WGS) and whole-exome sequencing (WXS) (Ishii, 2017).

Currently, SCNT is the main technique for the production of KI gene edited livestock (Table 3). Furthermore, about half of the published KO farm animals were produced by SCNT (Tables 1, 2). The primary advantage of this cell-mediated gene editing approach is the ability to verify that the gene-edited cells contain the desired genetic modification prior to live animal production takes place. This approach eliminates the occurrence of genetic mosaicism and has a potential to decrease the timeframe for generating the desired genotype and reducing the overall cost of animal production. These aspects are especially critical for application in large domestic animals that have particularly long generation intervals. While mosaicism resulting from CRISPR/Cas9 genome editing is typically regarded as an undesirable outcome, in certain cases, it may be valuable especially in animal models. These include assessments of candidate gene function *in vivo* where direct comparison of mutant and wild-type cells can be performed in the same organ of mosaic animals (Zhong et al., 2015). Mosaic animal models could also help us better understand the effect of gene dosage in congenital disorders. One example involves mosaicism of the Pax6 gene in mice. This gene plays an important role in eye development. CRISPR/Cas9-mediated mutation of Pax6 in mice have resulted in somatic mosaicism and variable developmental eye abnormalities in founder animals (Yasue et al., 2017). Thus, certain mosaic animal models could provide insights into the complexities of human congenital diseases that appear in mosaic form. Derivation of Bovine Embryonic Stem Cells (bESCs) was recently reported, and these cells could potentially be used as donor cells for nuclear transfer (Bogliotti et al., 2018). bESCs may offer some advantages compared to somatic cells such as greater *in vitro* longevity and potentially higher efficiency of homologous recombination. However, these hypothetical benefits will need to be further validated. Direct zygote manipulation, especially the zygote electroporation technique, is much less technically challenging compared to SCNT (Miao et al., 2019). Advancements in gene editing precision and efficiency, as well as developing strategies for reducing mosaicism have the potential to greatly enhance the accelerated and widespread utilization of gene editing technology in domestic animals, regardless of the specific application. This also assumes the technology receives favorable regulatory allowance, which will allow rapid integration of this high-value technology to contribute to the goal of increasing world-wide food security, and broad application as an important research tool.

## Author Contributions

IVP, ZF, and IP wrote the manuscript. All authors contributed to the review of appropriate literature, preparation, and review of the manuscript. All authors contributed to the article and approved the submitted version.

## Conflict of Interest

The authors declare that the research was conducted in the absence of any commercial or financial relationships that could be construed as a potential conflict of interest.

## References

[B282] Adli M. (2018). The CRISPR tool kit for genome editing and beyond. *Nat. Commun.* 9, 1–13. 29765029 10.1038/s41467-018-04252-2 PMC5953931

[B283] Aguzzi A. Baumann F. Bremer J. (2008). The prion’s elusive reason for being. *Annu. Rev. Neurosci.* 31, 439–477. 18558863 10.1146/annurev.neuro.31.060407.125620

[B284] Aida T. Nakade S. Sakuma T. Izu Y. Oishi A. Mochida K. (2016). Gene cassette knock-in in mammalian cells and zygotes by enhanced MMEJ. *BMC Genom.* 17, 1–18. 10.1186/s12864-016-3331-9 PMC5126809 27894274

[B1] Aird E. J. Lovendahl K. N. Martin A. S. Harris R. S. Gordon W. R. (2018). Increasing Cas9-mediated homology-directed repair efficiency through covalent tethering of DNA repair template. *Commun. Biol.* 1:54. 10.1038/s42003-018-0054-2 PMC6123678 30271937

[B2] Anzalone A. V. Koblan L. W. Liu D. R. (2020). Genome editing with CRISPR–Cas nucleases, base editors, transposases and prime editors. *Nat. Biotechnol.* 38 824–844. 10.1038/s41587-020-0561-9 32572269

[B3] Anzalone A. V. Randolph P. B. Davis J. R. Sousa A. A. Koblan L. W. Levy J. M. (2019). Search-and-replace genome editing without double-strand breaks or donor DNA. *Nature* 576 149–157. 10.1038/s41586-019-1711-4 31634902 PMC6907074

[B4] Baguisi A. Behboodi E. Melican D. T. Pollock J. S. Destrempes M. M. Cammuso C. (1999). Production of goats by somatic cell nuclear transfer. *Nat. Biotechnol.* 17 456–461. 10331804 10.1038/8632

[B5] Banan M. (2020). Recent advances in CRISPR/Cas9-mediated knock-ins in mammalian cells. *J. Biotechnol.* 308 1–9. 10.1016/j.jbiotec.2019.11.010 31751596

[B6] Bayna E. M. Rosen J. M. (1990). Tissue-specific, high level expression of the rat whey acidic protien gene in transgenic mice. *Nucleic Acids Res.* 18 2977–2985. 10.1093/nar/18.10.2977 2349094 PMC330827

[B7] Bi Y. Hua Z. Liu X. Hua W. Ren H. Xiao H. (2016). Isozygous and selectable marker-free MSTN knockout cloned pigs generated by the combined use of CRISPR/Cas9 and Cre/LoxP. *Sci. Rep.* 6:31729. 10.1038/srep31729 PMC4987667 27530319

[B8] Bibikova M. Carroll D. Segal D. J. Trautman J. K. Smith J. Kim Y.-G. (2001). Stimulation of homologous recombination through targeted cleavage by chimeric nucleases. *Mol. Cell. Biol.* 21 289–297. 10.1128/mcb.21.1.289-297.2001 11113203 PMC88802

[B9] Bischoff N. Wimberger S. Maresca M. Brakebusch C. (2020). Improving precise CRISPR genome editing by small molecules: is there a magic potion? *Cells* 9:1318 10.3390/cells9051318 PMC7291049 32466303

[B10] Bogdanovich S. Krag T. O. Barton E. R. Morris L. D. Whittemore L. A. Ahima R. S. (2002). Functional improvement of dystrophic muscle by myostatin blockade. *Nature* 420 418–421. 10.1038/nature01154 12459784

[B11] Bogliotti Y. S. Wu J. Vilarino M. Okamura D. Soto D. A. Zhong C. (2018). Efficient derivation of stable primed pluripotent embryonic stem cells from bovine blastocysts. *Proc. Natl. Acad. Sci. U. S. A.* 115 2090–2095. 10.1073/pnas.1716161115 29440377 PMC5834688

[B12] Boman I. A. Klemetsdal G. Blichfeldt T. Nafstad O. Vage D. I. (2009). A frameshift mutation in the coding region of the myostatin gene (MSTN) affects carcass conformation and fatness in Norwegian white sheep (Ovis aries). *Anim. Genet.* 40 418–422. 10.1111/j.1365-2052.2009.01855.x 19392824

[B13] Bueler H. Aguzzi A. Sailer A. Greiner R. A. Autenried P. Aguet M. (1993). Mice devoid of PrP are resistant to scrapie. *Cell* 73 1339–1347. 10.1016/0092-8674(93)90360-3 8100741

[B14] Cameron P. Fuller C. K. Donohoue P. D. Jones B. N. Thompson M. S. Carter M. M. (2017). Mapping the genomic landscape of CRISPR–Cas9 cleavage. *Nat. Methods* 14 600–606. 28459459 10.1038/nmeth.4284

[B15] Capecchi M. (1989). Altering the genome by homologous recombination. *Science* 244 1288–1292. 10.1126/science.2660260 2660260

[B16] Carey K. Ryu J. Uh K. Lengi A. J. Clark-Deener S. Corl B. A. (2019). Frequency of off-targeting in genome edited pigs produced via direct injection of the CRISPR/Cas9 system into developing embryos. *BMC Biotechnol.* 19:25. 10.1186/s12896-019-0517-7 PMC6501304 31060546

[B17] Carroll D. (2017). Focus: genome editing: genome editing: past, present, and future. *Yale J. Biol. Med.* 90:653. PMC5733845 29259529

[B18] Chandler M. De La Cruz F. Dyda F. Hickman A. B. Moncalian G. Ton-Hoang B. (2013). Breaking and joining single-stranded DNA: the HUH endonuclease superfamily. *Nat. Rev. Microbiol.* 11 525–538. 10.1038/nrmicro3067 23832240 PMC6493337

[B19] Chen F. Wang Y. Yuan Y. Zhang W. Ren Z. Jin Y. (2015). Generation of B cell-deficient pigs by highly efficient CRISPR/Cas9-mediated gene targeting. *J. Genet. Genomics* 42 437–444. 10.1016/j.jgg.2015.05.002 26336800

[B20] Chen J. An B. Yu B. Peng X. Yuan H. Yang Q. (2020). CRISPR/Cas9-mediated knockin of human factor IX into swine factor IX locus effectively alleviates bleeding in hemophilia B pigs. *Haematologica* 10.3324/haematol.2019.224063 Online ahead of print. 31974191 PMC7927883

[B21] Cho B. Kim S. J. Lee E.-J. Ahn S. M. Lee J. S. Ji D.-Y. (2018). Generation of insulin-deficient piglets by disrupting INS gene using CRISPR/Cas9 system. *Transgenic Res.* 27 289–300. 10.1007/s11248-018-0074-1 29691708

[B22] Cho S. W. Kim S. Kim Y. Kweon J. Kim H. S. Bae S. (2014). Analysis of off-target effects of CRISPR/Cas-derived RNA-guided endonucleases and nickases. *Genome Res.* 24 132–141. 10.1101/gr.162339.113 24253446 PMC3875854

[B23] Chuang C.-K. Chen C.-H. Huang C.-L. Su Y.-H. Peng S.-H. Lin T.-Y. (2017). Generation of GGTA1 mutant pigs by direct pronuclear microinjection of CRISPR/Cas9 plasmid vectors. *Anim. Biotechnol.* 28 174–181. 10.1080/10495398.2016.1246453 27834588

[B24] Cibelli J. B. Stice S. L. Golueke P. J. Kane J. J. Jerry J. Blackwell C. (1998). Cloned transgenic calves produced from nonquiescent fetal fibroblasts. *Science* 280 1256–1258. 10.1126/science.280.5367.1256 9596577

[B25] Ciccarelli M. Giassetti M. I. Miao D. Oatley M. J. Robbins C. Lopez-Biladeau B. (2020). Donor-derived spermatogenesis following stem cell transplantation in sterile NANOS2 knockout males. *Proc. Natl. Acad. Sci*. 117 24195–24204. 10.1073/pnas.2010102117 32929012 PMC7533891

[B26] Clark A. J. Burl S. Denning C. Dickinson P. (2000). Gene targeting in livestock: a preview. *Transgenic Res.* 9 263–275. 11131006 10.1023/a:1008974616402

[B27] Clark J. Whitelaw B. (2003). A future for transgenic livestock. *Nat. Rev. Genet.* 4 825–833. 14526378 10.1038/nrg1183 PMC7097355

[B28] Coelho M. A. De Braekeleer E. Firth M. Bista M. Lukasiak S. Cuomo M. E. (2020). CRISPR GUARD protects off-target sites from Cas9 nuclease activity using short guide RNAs. *Nat. Commun.* 11:4132. 10.1038/s41467-020-17952-5 PMC7431537 32807781

[B29] Cong L. Ran F. A. Cox D. Lin S. Barretto R. Habib N. (2013). Multiplex genome engineering using CRISPR/Cas systems. *Science* 339 819–823. 23287718 10.1126/science.1231143 PMC3795411

[B30] Conlon D. L. A. R. (2006). Animal models for disease: knockout, knockin and conditional mutant mice. *Methods Mol. Med.* 129 41–67. 10.1385/1-59745-213-0:41 17085804

[B31] Cowan P. J. Hawthorne W. J. Nottle M. B. (2019). Xenogeneic transplantation and tolerance in the era of CRISPR-Cas9. *Curr. Opin. Organ. Transplant.* 24 5–11. 10.1097/mot.0000000000000589 30480643

[B32] Crispo M. Mulet A. Tesson L. Barrera N. Cuadro F. Dos Santos-Neto P. (2015). Efficient generation of myostatin knock-out sheep using CRISPR/Cas9 technology and microinjection into zygotes. *PLoS One* 10:e0136690. 10.1371/journal.pone.0136690 26305800 PMC4549068

[B33] Dai Y. Vaught T. Boone J. Chen S. Phelps C. Ball S. (2002). Targeted disruption of the alpha 1,3-galactosyltransferase gene in cloned pigs. *Nat. Biotechnol.* 20 251–255. 10.1038/nbt0302-251 11875425

[B34] Davis L. Maizels N. (2016). Two distinct pathways support gene correction by single-stranded donors at DNA nicks. *Cell Rep.* 17 1872–1881. 10.1016/j.celrep.2016.10.049 27829157 PMC5108528

[B35] Deng S. Kongpan L. Wang F. Ning L. Liu G. Zhao Y. (2014). One-step generation of myostatin gene knockout sheep via the CRISPR/Cas9 system. *Front. Agr. Sci. Eng.* 1:2–5. 10.15302/j-fase-2014007

[B36] Derscheid R. J. Ackermann M. R. (2012). Perinatal lamb model of respiratory syncytial virus (RSV) infection. *Viruses* 4 2359–2378. 10.3390/v4102359 23202468 PMC3497056

[B37] DeWitt M. A. Corn J. E. Carroll D. (2017). Genome editing via delivery of Cas9 ribonucleoprotein. *Methods* 121 9–15. 10.1016/j.ymeth.2017.04.003 28410976 PMC6698184

[B38] Dow L. E. (2015). Modeling disease in vivo with CRISPR/Cas9. *Trends Mol. Med.* 21 609–621. 10.1016/j.molmed.2015.07.006 26432018 PMC4592741

[B39] Eaton S. L. Proudfoot C. Lillico S. Skehel P. Kline R. Hamer K. (2019). CRISPR/Cas9 mediated generation of an ovine model for infantile neuronal ceroid lipofuscinosis (CLN1 disease). *Sci. Rep.* 9:9891. 10.1038/s41598-019-45859-9 PMC6616324 31289301

[B40] Fan Z. Perisse I. V. Cotton C. U. Regouski M. Meng Q. Domb C. (2018a). A sheep model of cystic fibrosis generated by CRISPR/Cas9 disruption of the CFTR gene. *JCI Insight* 3:e123529. 10.1172/jci.insight.123529 PMC6237476 30282831

[B41] Fan Z. Regouski M. Van Wettere A. Wang Z. Sullivan E. Polejaeva I. (2018b). 28 generation of immunoglobulin heavy constant mu (IGHM) knockout goats using CRISPR/Cas9 and somatic cell nuclear transfer. *Reprod. Fertil. Dev.* 30 153–154. 10.1071/rdv30n1ab28

[B42] Fang B. Ren X. Wang Y. Li Z. Zhao L. Zhang M. (2018). Apolipoprotein E deficiency accelerates atherosclerosis development in miniature pigs. *Dis. Models Mech.* 11:dmm036632. 10.1242/dmm.036632 30305304 PMC6215431

[B43] Fischer K. Kraner-Scheiber S. Petersen B. Rieblinger B. Buermann A. Flisikowska T. (2016). Efficient production of multi-modified pigs for xenotransplantation by ‘combineering’, gene stacking and gene editing. *Sci. Rep.* 6:29081. 10.1038/srep29081 PMC4926246 27353424

[B44] Foley J. A. Ramankutty N. Brauman K. A. Cassidy E. S. Gerber J. S. Johnston M. (2011). Solutions for a cultivated planet. *Nature* 478 337–342. 21993620 10.1038/nature10452

[B45] Gaj T. Gersbach C. A. Barbas C. F. I. I. I. (2013). ZFN, TALEN, and CRISPR/Cas-based methods for genome engineering. *Trends Biotechnol.* 31 397–405. 10.1016/j.tibtech.2013.04.004 23664777 PMC3694601

[B46] Gao H. Zhao C. Xiang X. Li Y. Zhao Y. Li Z. (2016). Production of α1, 3-galactosyltransferase and cytidine monophosphate-N-acetylneuraminic acid hydroxylase gene double-deficient pigs by CRISPR/Cas9 and handmade cloning. *J. Reprod. Dev.* 63 17–26. 10.1262/jrd.2016-079 27725344 PMC5320426

[B47] Gao Y. Wu H. Wang Y. Liu X. Chen L. Li Q. (2017). Single Cas9 nickase induced generation of NRAMP1 knockin cattle with reduced off-target effects. *Genome Biol.* 18:13. 10.1186/s13059-016-1144-4 PMC5286826 28143571

[B48] Gaudelli N. M. Komor A. C. Rees H. A. Packer M. S. Badran A. H. Bryson D. I. (2017). Programmable base editing of A.T to G.C in genomic DNA without DNA cleavage. *Nature* 551 464–471. 10.1038/nature24644 29160308 PMC5726555

[B49] Georges M. Charlier C. Hayes B. (2019). Harnessing genomic information for livestock improvement. *Nat. Rev. Genet.* 20 135–156. 10.1038/s41576-018-0082-2 30514919

[B50] Gilbert L. A. Larson M. H. Morsut L. Liu Z. Brar G. A. Torres S. E. (2013). CRISPR-mediated modular RNA-guided regulation of transcription in eukaryotes. *Cell* 154 442–451. 10.1016/j.cell.2013.06.044 23849981 PMC3770145

[B51] Godfray H. C. Beddington J. R. Crute I. R. Haddad L. Lawrence D. Muir J. F. (2010). Food security: the challenge of feeding 9 billion people. *Science* 327 812–818. 20110467 10.1126/science.1185383

[B52] Gordon J. W. Scangos G. A. Plotkin D. J. Barbosa J. A. Ruddle F. H. (1980). Genetic transformation of mouse embryos by microinjection of purified DNA. *Proc. Natl. Acad. Sci. U. S. A.* 77 7380–7384. 10.1073/pnas.77.12.7380 6261253 PMC350507

[B53] Grobet L. Martin L. J. Poncelet D. Pirottin D. Brouwers B. Riquet J. (1997). A deletion in the bovine myostatin gene causes the double-muscled phenotype in cattle. *Nat. Genet.* 17 71–74. 10.1038/ng0997-71 9288100

[B54] Grobet L. Poncelet D. Royo L. J. Brouwers B. Pirottin D. Michaux C. (1998). Molecular definition of an allelic series of mutations disrupting the myostatin function and causing double-muscling in cattle. *Mamm. Genome* 9 210–213. 10.1007/s003359900727 9501304

[B55] Grünewald J. Zhou R. Iyer S. Lareau C. A. Garcia S. P. Aryee M. J. (2019). CRISPR DNA base editors with reduced RNA off-target and self-editing activities. *Nat. Biotechnol.* 37 1041–1048. 10.1038/s41587-019-0236-6 31477922 PMC6730565

[B56] Guo R. Wan Y. Xu D. Cui L. Deng M. Zhang G. (2016). Generation and evaluation of Myostatin knock-out rabbits and goats using CRISPR/Cas9 system. *Sci. Rep.* 6:29855. 10.1038/srep29855 PMC4945924 27417210

[B57] Gutierrez K. Dicks N. Glanzner W. G. Agellon L. B. Bordignon V. (2015). Efficacy of the porcine species in biomedical research. *Front. Genet.* 6:293. 10.3389/fgene.2015.00293 PMC4584988 26442109

[B58] Haeussler M. (2020). CRISPR off-targets: a question of context. *Cell. Biol. Toxicol.* 36 5–9. 10.1007/s10565-019-09497-1 31734746 PMC7056574

[B59] Hai T. Guo W. Yao J. Cao C. Luo A. Qi M. (2017). Creation of miniature pig model of human Waardenburg syndrome type 2A by ENU mutagenesis. *Hum. Genet.* 136 1463–1475. 10.1007/s00439-017-1851-2 29094203

[B60] Hai T. Teng F. Guo R. Li W. Zhou Q. (2014). One-step generation of knockout pigs by zygote injection of CRISPR/Cas system. *Cell Res.* 24 372–375. 10.1038/cr.2014.11 24481528 PMC3945887

[B61] Hamernik D. L. (2019). Farm animals are important biomedical models. *Anim. Front.* 9 3–5. 10.1093/af/vfz026 32002256 PMC6951888

[B62] Hammer R. E. Pursel V. G. Rexroad C. E. Wall R. J. Bolt D. J. Ebert K. M. (1985). Production of transgenic rabbits, sheep and pigs by microinjection. *Nature* 315 680–683. 10.1038/315680a0 3892305

[B63] Han K. Liang L. Li L. Ouyang Z. Zhao B. Wang Q. (2017). Generation of Hoxc13 knockout pigs recapitulates human ectodermal dysplasia–9. *Hum. Mol. Genet.* 26 184–191. 28011715 10.1093/hmg/ddw378

[B64] Hao F. Yan W. Li X. Wang H. Wang Y. Hu X. (2018). Generation of cashmere goats carrying an EDAR gene mutant using CRISPR-Cas9-mediated genome editing. *Intl. J. Biol. Sci.* 14 427–436. 10.7150/ijbs.23890 29725264 PMC5930475

[B65] Harmsen T. Klaasen S. Van De Vrugt H. Te Riele H. (2018). DNA mismatch repair and oligonucleotide end-protection promote base-pair substitution distal from a CRISPR/Cas9-induced DNA break. *Nucleic Acids Res.* 46 2945–2955. 10.1093/nar/gky076 29447381 PMC5888797

[B66] Harrison P. T. Hart S. (2018). A beginner’s guide to gene editing. *Exp. Physiol.* 103 439–448. 29282799 10.1113/EP086047

[B67] Hashimoto M. Yamashita Y. Takemoto T. (2016). Electroporation of Cas9 protein/sgRNA into early pronuclear zygotes generates non-mosaic mutants in the mouse. *Dev. Biol.* 418 1–9. 10.1016/j.ydbio.2016.07.017 27474397

[B68] He Z. Zhang T. Jiang L. Zhou M. Wu D. Mei J. (2018). Use of CRISPR/Cas9 technology efficiently targetted goat myostatin through zygotes microinjection resulting in double-muscled phenotype in goats. *Biosci. Rep.* 38:BSR20180742. 10.1042/BSR20180742 PMC6239268 30201688

[B69] Hendel A. Bak R. O. Clark J. T. Kennedy A. B. Ryan D. E. Roy S. (2015). Chemically modified guide RNAs enhance CRISPR-Cas genome editing in human primary cells. *Nat. Biotechnol.* 33 985–989. 10.1038/nbt.3290 26121415 PMC4729442

[B70] Hoellerbauer P. Kufeld M. Paddison P. J. (2020). efficient multi-allelic genome editing of primary cell cultures via CRISPR-Cas9 ribonucleoprotein nucleofection. *Curr. Protoc. Stem Cell Biol.* 54:e126. 10.1002/cpsc.126 32833346

[B71] Hsu P. D. Lander E. S. Zhang F. (2014). Development and applications of CRISPR-Cas9 for genome engineering. *Cell* 157 1262–1278. 10.1016/j.cell.2014.05.010 24906146 PMC4343198

[B72] Hu J. H. Miller S. M. Geurts M. H. Tang W. Chen L. Sun N. (2018). Evolved Cas9 variants with broad PAM compatibility and high DNA specificity. *Nature* 556 57–63. 10.1038/nature26155 29512652 PMC5951633

[B73] Hu R. Fan Z. Wang B. Deng S. Zhang X. Zhang J. (2017). RAPID communication: generation of FGF5 knockout sheep via the CRISPR/Cas9 system. *J. Anim. Sci.* 95 2019–2024. 10.2527/jas.2017.1503 28727005

[B74] Huang J. Wang A. Huang C. Sun Y. Song B. Zhou R. (2020). Generation of marker-free pbd-2 KNock-in pigs using the CRISPR/Cas9 and Cre/loxP systems. *Genes* 11:951 10.3390/genes11080951 PMC7465224 32824735

[B75] Huang L. Hua Z. Xiao H. Cheng Y. Xu K. Gao Q. (2017). CRISPR/Cas9-mediated ApoE-/-and LDLR-/-double gene knockout in pigs elevates serum LDL-C and TC levels. *Oncotarget* 8 37751–37760. 10.18632/oncotarget.17154 28465483 PMC5514946

[B76] Humphrey S. E. Kasinski A. L. (2015). RNA-guided CRISPR-Cas technologies for genome-scale investigation of disease processes. *J. Hematol. Oncol.* 8:31. 10.1186/s13045-015-0127-3 PMC4389696 25888285

[B77] Iiizumi S. Nomura Y. So S. Uegaki K. Aoki K. Shibahara K.-I. (2006). Simple one-week method to construct gene-targeting vectors: application to production of human knockout cell lines. *Biotechniques* 41 311–316. 10.2144/000112233 16989091

[B78] Ikeda M. Matsuyama S. Akagi S. Ohkoshi K. Nakamura S. Minabe S. (2017). Correction of a disease mutation using CRISPR/Cas9-assisted genome editing in Japanese black cattle. *Sci. Rep.* 7:17827. 10.1038/s41598-017-17968-w PMC5736618 29259316

[B79] Ishii T. (2017). Genome-edited livestock: ethics and social acceptance. *Anim. Front.* 7 24–32. 10.2527/af.2017.0115 32704858

[B80] Jiang F. Doudna J. A. (2017). CRISPR–Cas9 structures and mechanisms. *Annu. Rev. Biophys.* 46 505–529. 10.1146/annurev-biophys-062215-010822 28375731

[B81] Jiang F. Taylor D. W. Chen J. S. Kornfeld J. E. Zhou K. Thompson A. J. (2016). Structures of a CRISPR-Cas9 R-loop complex primed for DNA cleavage. *Science* 351 867–871. 10.1126/science.aad8282 26841432 PMC5111852

[B82] Jin Y.-H. Joo H. Lee K. Kim H. Didier R. Yang Y. (2019). Streamlined procedure for gene knockouts using all-in-one adenoviral CRISPR-Cas9. *Sci. Rep.* 9:277. 10.1038/s41598-018-36736-y PMC6342919 30670765

[B83] Joanna Z. Magdalena H. Agnieszka N.-T. Jacek J. Ryszard S. Zdzisław S. (2018). The production of UL16-binding protein 1 targeted pigs using CRISPR technology. *3 Biotech* 8:70. 10.1007/s13205-018-1107-4 PMC5766454 29354381

[B84] Johnson R. D. Jasin M. (2000). Sister chromatid gene conversion is a prominent double-strand break repair pathway in mammalian cells. *EMBO J.* 19 3398–3407. 10.1093/emboj/19.13.3398 10880452 PMC313931

[B85] Joung J. K. Sander J. D. (2013). TALENs: a widely applicable technology for targeted genome editing. *Nat. Rev. Mol. Cell Biol.* 14 49–55. 10.1038/nrm3486 23169466 PMC3547402

[B86] Kalds P. Gao Y. Zhou S. Cai B. Huang X. Wang X. (2020). Redesigning small ruminant genomes with CRISPR toolkit: overview and perspectives. *Theriogenology* 147 25–33. 10.1016/j.theriogenology.2020.02.015 32086048

[B87] Kalds P. Zhou S. Cai B. Liu J. Wang Y. Petersen B. (2019). Sheep and goat genome engineering: from random transgenesis to the CRISPR era. *Front. Genet.* 10:750. 10.3389/fgene.2019.00750 PMC6735269 31552084

[B88] Kan Y. Ruis B. Takasugi T. Hendrickson E. A. (2017). Mechanisms of precise genome editing using oligonucleotide donors. *Genome Res.* 27 1099–1111. 10.1101/gr.214775.116 28356322 PMC5495063

[B89] Kang J. T. Ryu J. Cho B. Lee E. J. Yun Y. J. Ahn S. (2016b). Generation of RUNX 3 knockout pigs using CRISPR/Cas9-mediated gene targeting. *Reprod. Domest. Anim.* 51 970–978. 27696566 10.1111/rda.12775

[B90] Kang J.-T. Cho B. Ryu J. Ray C. Lee E.-J. Yun Y.-J. (2016a). Biallelic modification of IL2RG leads to severe combined immunodeficiency in pigs. *Reprod. Biol. Endocrinol.* 14:74. 10.1186/s12958-016-0206-5 PMC5095964 27809915

[B91] Kelly J. J. Saee-Marand M. Nyström N. N. Chen Y. Evans M. M. Hamilton A. M. (2020). A safe harbor-targeted CRISPR/Cas9 homology independent targeted integration (HITI) system for multi-modality reporter gene-based cell tracking. *bioRxiv [preprint].* 10.1101/2020.02.10.942672 PMC7817109 33523917

[B92] Khan A. J. Misenko S. M. Thandoni A. Schiff D. Jhawar S. R. Bunting S. F. (2018). VX-984 is a selective inhibitor of non-homologous end joining, with possible preferential activity in transformed cells. *Oncotarget* 9 25833–25841. 10.18632/oncotarget.25383 29899825 PMC5995231

[B93] Khan S. H. (2019). Genome-editing technologies: concept, pros, and cons of various genome-editing techniques and bioethical concerns for clinical application. *Mol. Ther.Nucleic Acids* 16 326–334. 10.1016/j.omtn.2019.02.027 30965277 PMC6454098

[B94] Kim D. Bae S. Park J. Kim E. Kim S. Yu H. R. (2015). Digenome-seq: genome-wide profiling of CRISPR-Cas9 off-target effects in human cells. *Nat. Methods* 12 237–243. 10.1038/nmeth.3284 25664545

[B95] Kim H. S. Jeong Y. K. Hur J. K. Kim J.-S. Bae S. (2019). Adenine base editors catalyze cytosine conversions in human cells. *Nat. Biotechnol.* 37 1145–1148. 10.1038/s41587-019-0254-4 31548727

[B96] Kim K. Ryu S.-M. Kim S.-T. Baek G. Kim D. Lim K. (2017). Highly efficient RNA-guided base editing in mouse embryos. *Nat. Biotechnol.* 35 435–437. 10.1038/nbt.3816 28244995

[B97] Kim S. Kim D. Cho S. W. Kim J. Kim J. S. (2014). Highly efficient RNA-guided genome editing in human cells via delivery of purified Cas9 ribonucleoproteins. *Genome Res.* 24 1012–1019. 10.1101/gr.171322.113 24696461 PMC4032847

[B98] Kim T. K. Eberwine J. H. (2010). Mammalian cell transfection: the present and the future. *Anal. Bioanal. Chem.* 397 3173–3178. 10.1007/s00216-010-3821-6 20549496 PMC2911531

[B99] Kim Y. B. Komor A. C. Levy J. M. Packer M. S. Zhao K. T. Liu D. R. (2017). Increasing the genome-targeting scope and precision of base editing with engineered Cas9-cytidine deaminase fusions. *Nat. Biotechnol.* 35 371–376. 10.1038/nbt.3803 28191901 PMC5388574

[B100] Koblan L. W. Doman J. L. Wilson C. Levy J. M. Tay T. Newby G. A. (2018). Improving cytidine and adenine base editors by expression optimization and ancestral reconstruction. *Nat. Biotechnol.* 36 843–846. 10.1038/nbt.4172 29813047 PMC6126947

[B101] Kohn D. Kantoff P. Eglitis M. Mclachlin J. Moen R. Karson E. (1987). Retroviral-mediated gene transfer into mammalian cells. *Blood cells* 13 285–298. 3311223

[B102] Komor A. C. Kim Y. B. Packer M. S. Zuris J. A. Liu D. R. (2016). Programmable editing of a target base in genomic DNA without double-stranded DNA cleavage. *Nature* 533 420–424. 10.1038/nature17946 27096365 PMC4873371

[B103] Komor A. C. Zhao K. T. Packer M. S. Gaudelli N. M. Waterbury A. L. Koblan L. W. (2017). Improved base excision repair inhibition and bacteriophage Mu Gam protein yields C: G-to-T: a base editors with higher efficiency and product purity. *Sci. Adv.* 3:eaao4774. 10.1126/sciadv.aao4774 28875174 PMC5576876

[B104] Kostyushev D. Kostyusheva A. Brezgin S. Zarifyan D. Utkina A. Goptar I. (2019). Suppressing the NHEJ pathway by DNA-PKcs inhibitor NU7026 prevents degradation of HBV cccDNA cleaved by CRISPR/Cas9. *Sci. Rep.* 9:1847. 10.1038/s41598-019-38526-6 PMC6372644 30755668

[B105] Kurt I. C. Zhou R. Iyer S. Garcia S. P. Miller B. R. Langner L. M. (2020). CRISPR C-to-G base editors for inducing targeted DNA transversions in human cells. *Nat. Biotechnol.* 10.1038/s41587-020-0609-x [Epub ahead of print]. 32690971 PMC7854778

[B106] Lai S. Wei S. Zhao B. Ouyang Z. Zhang Q. Fan N. (2016). Generation of knock-in pigs carrying Oct4-tdTomato reporter through CRISPR/Cas9-mediated genome engineering. *PLoS One* 11:e0146562. 10.1371/journal.pone.0146562 26756580 PMC4710570

[B107] Laible G. Wei J. Wagner S. (2015). Improving livestock for agriculture - technological progress from random transgenesis to precision genome editing heralds a new era. *Biotechnol. J.* 10 109–120. 10.1002/biot.201400193 25515661

[B108] Lamas-Toranzo I. Galiano-Cogolludo B. Cornudella-Ardiaca F. Cobos-Figueroa J. Ousinde O. Bermejo-Alvarez P. (2019). Strategies to reduce genetic mosaicism following CRISPR-mediated genome edition in bovine embryos. *Sci. Rep.* 9:14900. 10.1038/s41598-019-51366-8 PMC6797768 31624292

[B109] Lawhorn I. E. Ferreira J. P. Wang C. L. (2014). Evaluation of sgRNA target sites for CRISPR-mediated repression of TP53. *PLoS One* 9:e113232. 10.1371/journal.pone.0113232 25398078 PMC4232525

[B110] Lee K. Uh K. Farrell K. (2020). Current progress of genome editing in livestock. *Theriogenology* 150 229–235. 10.1016/j.theriogenology.2020.01.036 32000993 PMC7234903

[B111] Lee S. J. (2004). Regulation of muscle mass by myostatin. *Annu. Rev. Cell Dev. Biol.* 20 61–86. 10.1146/annurev.cellbio.20.012103.135836 15473835

[B112] Lee S. J. Reed L. A. Davies M. V. Girgenrath S. Goad M. E. Tomkinson K. N. (2005). Regulation of muscle growth by multiple ligands signaling through activin type II receptors. *Proc. Natl. Acad. Sci. U.S.A.* 102 18117–18122. 10.1073/pnas.0505996102 16330774 PMC1306793

[B113] Lei S. Ryu J. Wen K. Twitchell E. Bui T. Ramesh A. (2016). Increased and prolonged human norovirus infection in RAG2/IL2RG deficient gnotobiotic pigs with severe combined immunodeficiency. *Sci. Rep.* 6:25222. 10.1038/srep25222 PMC4846862 27118081

[B114] Li G. Zhang X. Wang H. Mo J. Zhong C. Shi J. (2020). CRISPR/Cas9-mediated integration of large transgene into pig CEP112 locus. *G3*, 10 467–473. 10.1534/g3.119.400810 31818875 PMC7003105

[B115] Li G. Zhou S. Li C. Cai B. Yu H. Ma B. (2019). Base pair editing in goat: nonsense codon introgression into FGF 5 results in longer hair. *FEBS J.* 286 4675–4692. 10.1111/febs.14983 31276295

[B116] Li P. Estrada J. L. Burlak C. Montgomery J. Butler J. R. Santos R. M. (2015). Efficient generation of genetically distinct pigs in a single pregnancy using multiplexed single-guide RNA and carbohydrate selection. *Xenotransplantation* 22 20–31. 10.1111/xen.12131 25178170

[B117] Li R. Zeng W. Ma M. Wei Z. Liu H. Liu X. (2020). Precise editing of myostatin signal peptide by CRISPR/Cas9 increases the muscle mass of Liang Guang Small Spotted pigs. *Transgenic Res.* 29 149–163. 10.1007/s11248-020-00188-w 31927726

[B118] Li W. R. Liu C. X. Zhang X. M. Chen L. Peng X. R. He S. G. (2017). CRISPR/Cas9-mediated loss of FGF5 function increases wool staple length in sheep. *FEBS J.* 284 2764–2773. 10.1111/febs.14144 28631368

[B119] Li X. Hao F. Hu X. Wang H. Dai B. Wang X. (2019). Generation of Tβ4 knock-in Cashmere goat using CRISPR/Cas9. *Intl. J. Biol. Sci.* 15 1743–1754. 10.7150/ijbs.34820 31360116 PMC6643211

[B120] Li Z. Yang H.-Y. Wang Y. Zhang M.-L. Liu X.-R. Xiong Q. (2017). Generation of tryptophan hydroxylase 2 gene knockout pigs by CRISPR/Cas9-mediated gene targeting. *J. Biomed. Res.* 31 445–452. 28866660 10.7555/JBR.31.20170026 PMC5706437

[B121] Liggins G. C. Howie R. N. (1972). A controlled trial of antepartum glucocorticoid treatment for prevention of the respiratory distress syndrome in premature infants. *Pediatrics* 50 515–525. 4561295

[B122] Lillico S. G. Proudfoot C. King T. J. Tan W. F. Zhang L. Mardjuki R. (2016). Mammalian interspecies substitution of immune modulatory alleles by genome editing. *Sci. Rep.* 6:21645. 10.1038/srep21645 PMC4761920 26898342

[B123] Liu Y. Li X. He S. Huang S. Li C. Chen Y. (2020). Efficient generation of mouse models with the prime editing system. *Cell Discov.* 6:27. 10.1038/s41421-020-0165-z PMC7186222 32351707

[B124] Liu Z. Chen S. Shan H. Jia Y. Chen M. Song Y. (2020). Efficient base editing with high precision in rabbits using YFE-BE4max. *Cell Death Dis.* 11:36. 10.1038/s41419-020-2244-3 PMC6971250 31959743

[B125] Liu Z. Lu Z. Yang G. Huang S. Li G. Feng S. (2018). Efficient generation of mouse models of human diseases via ABE-and BE-mediated base editing. *Nat. Commun.* 9:2338. 10.1038/s41467-018-04768-7 PMC6002399 29904106

[B126] Lovendahl Klaus (2018). Adaptation of HUH endonucleases for protein-DNA conjugation. Retrieved from the University of Minnesota Digital Conservancy. Available online at: http://hdl.handle.net/11299/201685 (accessed September 15, 2020).

[B127] Manson J. C. Clarke A. R. Hooper M. L. Aitchison L. Mcconnell I. Hope J. (1994). 129/Ola mice carrying a null mutation in PrP that abolishes mRNA production are developmentally normal. *Mol. Neurobiol.* 8 121–127. 10.1007/bf02780662 7999308

[B128] Marraffini L. A. (2015). CRISPR-Cas immunity in prokaryotes. *Nature* 526 55–61. 10.1038/nature15386 26432244

[B129] Maruyama T. Dougan S. K. Truttmann M. C. Bilate A. M. Ingram J. R. Ploegh H. L. (2015). Increasing the efficiency of precise genome editing with CRISPR-Cas9 by inhibition of nonhomologous end joining. *Nat. Biotechnol.* 33 538–542. 10.1038/nbt.3190 25798939 PMC4618510

[B130] McCreath K. J. Howcroft J. Campbell K. H. Colman A. Schnieke A. E. Kind A. J. (2000). Production of gene-targeted sheep by nuclear transfer from cultured somatic cells. *Nature* 405 1066–1069. 10.1038/35016604 10890449

[B131] McFarlane G. R. Salvesen H. A. Sternberg A. Lillico S. G. (2019). On-farm livestock genome editing using cutting edge reproductive technologies. *Front. Sustain. Food Syst.* 3:106 10.3389/fsufs

[B132] McMahon M. A. Rahdar M. Porteus M. (2012). Gene editing: not just for translation anymore. *Nat. Methods* 9 28–31. 10.1038/nmeth.1811 22205513

[B133] McMichael A. J. (2012). Insights from past millennia into climatic impacts on human health and survival. *Proc. Natl. Acad. Sci. U. S. A.* 109 4730–4737. 10.1073/pnas.1120177109 22315419 PMC3324023

[B134] McPherron A. C. Lawler A. M. Lee S. J. (1997). Regulation of skeletal muscle mass in mice by a new TGF-beta superfamily member. *Nature* 387 83–90. 10.1038/387083a0 9139826

[B135] McVey M. Lee S. E. (2008). MMEJ repair of double-strand breaks (director’s cut): deleted sequences and alternative endings. *Trends Genet.* 24 529–538. 10.1016/j.tig.2008.08.007 18809224 PMC5303623

[B136] Mehravar M. Shirazi A. Nazari M. Banan M. (2019). Mosaicism in CRISPR/Cas9-mediated genome editing. *Dev. Biol.* 445 156–162. 10.1016/j.ydbio.2018.10.008 30359560

[B137] Mei Y. Wang Y. Chen H. Sun Z. S. Ju X.-D. (2016). Recent progress in CRISPR/Cas9 technology. *J. Genet. Genom.* 43 63–75. 10.1016/j.jgg.2016.01.001 26924689

[B138] Meier R. P. H. Muller Y. D. Balaphas A. Morel P. Pascual M. Seebach J. D. (2018). Xenotransplantation: back to the future? *Transpl. Int.* 31 465–477. 10.1111/tri.13104 29210109

[B139] Menchaca A. Dos Santos-Neto P. Mulet A. Crispo M. (2020a). CRISPR in livestock: from editing to printing. *Theriogenology* 150 247–254. 10.1016/j.theriogenology.2020.01.063 32088034 PMC7102594

[B140] Menchaca A. Dos Santos-Neto P. Souza-Neves M. Cuadro F. Mulet A. Tesson L. (2020b). Otoferlin gene editing in sheep via cRiSpR-assisted ssoDn-mediated homology directed repair. *Sci. Rep.* 10:5995. 10.1038/s41598-020-62879-y PMC7138848 32265471

[B141] Meyer M. De Angelis M. H. Wurst W. Kühn R. (2010). Gene targeting by homologous recombination in mouse zygotes mediated by zinc-finger nucleases. *Proc. Natl. Acad. Sci. U. S. A.* 107 15022–15026. 10.1073/pnas.1009424107 20686113 PMC2930558

[B142] Miao D. Giassetti M. I. Ciccarelli M. Lopez-Biladeau B. Oatley J. M. (2019). Simplified pipelines for genetic engineering of mammalian embryos by CRISPR-Cas9 electroporationdagger. *Biol. Reprod.* 101 177–187. 10.1093/biolre/ioz075 31095680 PMC6614578

[B143] Mojica F. J. Díez-Villaseñor C. Soria E. Juez G. (2000). Biological significance of a family of regularly spaced repeats in the genomes of Archaea, bacteria and mitochondria. *Mol. Microbiol.* 36 244–246. 10.1046/j.1365-2958.2000.01838.x 10760181

[B144] Mosher D. S. Quignon P. Bustamante C. D. Sutter N. B. Mellersh C. S. Parker H. G. (2007). A mutation in the myostatin gene increases muscle mass and enhances racing performance in heterozygote dogs. *PLoS Genet.* 3:e79 10.1371/journal.pgen.0030079.eor PMC1877876 17530926

[B145] Nakade S. Tsubota T. Sakane Y. Kume S. Sakamoto N. Obara M. (2014). Microhomology-mediated end-joining-dependent integration of donor DNA in cells and animals using TALENs and CRISPR/Cas9. *Nat. Commun.* 5:5560. 10.1038/ncomms6560 PMC4263139 25410609

[B146] Navarro-Serna S. Vilarino M. Park I. Gadea J. Ross P. J. (2020). Livestock gene editing by one-step embryo manipulation. *J. Equ. Vet. Sci.* 89:103025. 10.1016/j.jevs.2020.103025 32563448

[B147] Nelson A. Tompkins K. Ramirez M. P. Gordon W. R. (2019). Interrogating mechanisms of ssDNA binding to a viral HUH-endonuclease by alanine scanning of an electrostatic patch. *bioRxiv [Preprint]* 10.1101/861070

[B148] Ni W. Qiao J. Hu S. Zhao X. Regouski M. Yang M. (2014). Efficient gene knockout in goats using CRISPR/Cas9 system. *PLoS One* 9:e106718. 10.1371/journal.pone.0106718 25188313 PMC4154755

[B149] Nishida K. Arazoe T. Yachie N. Banno S. Kakimoto M. Tabata M. (2016). Targeted nucleotide editing using hybrid prokaryotic and vertebrate adaptive immune systems. *Science* 353:aaf8729. 10.1126/science.aaf8729 27492474

[B150] Niu D. Wei H.-J. Lin L. George H. Wang T. Lee I.-H. (2017). Inactivation of porcine endogenous retrovirus in pigs using CRISPR-Cas9. *Science* 357 1303–1307. 10.1126/science.aan4187 28798043 PMC5813284

[B151] Niu Y. Jin M. Li Y. Li P. Zhou J. Wang X. (2017). Biallelic β-carotene oxygenase 2 knockout results in yellow fat in sheep via CRISPR/Cas9. *Anim. Genet.* 48 242–244. 10.1111/age.12515 27862083

[B152] Niu Y. Zhao X. Zhou J. Li Y. Huang Y. Cai B. (2018). Efficient generation of goats with defined point mutation (I397V) in GDF9 through CRISPR/Cas9. *Reprod. Fertil. Dev.* 30 307–312. 10.1071/rd17068 28692815

[B153] Nottle M. B. Salvaris E. J. Fisicaro N. Mcilfatrick S. Vassiliev I. Hawthorne W. J. (2017). Targeted insertion of an anti-CD2 monoclonal antibody transgene into the GGTA1 locus in pigs using Fok I-dCas9. *Sci. Rep.* 7:8383. 10.1038/s41598-017-09030-6 PMC5559588 28814758

[B154] O’Brien A. R. Wilson L. O. Burgio G. Bauer D. C. (2019). Unlocking HDR-mediated nucleotide editing by identifying high-efficiency target sites using machine learning. *Sci. Rep.* 9:2788. 10.1038/s41598-019-39142-0 PMC6391469 30808944

[B155] Oceguera-Yanez F. Kim S.-I. Matsumoto T. Tan G. W. Xiang L. Hatani T. (2016). Engineering the AAVS1 locus for consistent and scalable transgene expression in human iPSCs and their differentiated derivatives. *Methods* 101 43–55. 10.1016/j.ymeth.2015.12.012 26707206

[B156] Okamoto S. Amaishi Y. Maki I. Enoki T. Mineno J. (2019). Highly efficient genome editing for single-base substitutions using optimized ssODNs with Cas9-RNPs. *Sci. Rep.* 9:4811. 10.1038/s41598-019-41121-4 PMC6423289 30886178

[B157] O’Neil E. V. Brooks K. Burns G. W. Ortega M. S. Denicol A. C. Aguiar L. H. (2020). Prostaglandin-endoperoxide synthase 2 is not required for preimplantation ovine conceptus development in sheep. *Mol. Reprod. Dev.* 87 142–151. 10.1002/mrd.23300 31746519

[B158] Ostedgaard L. S. Price M. P. Whitworth K. M. Abou Alaiwa M. H. Fischer A. J. Warrier A. (2020). Lack of airway submucosal glands impairs respiratory host defenses. *Elife* 9:e59653. 10.7554/eLife.59653 PMC7541087 33026343

[B159] Paix A. Folkmann A. Goldman D. H. Kulaga H. Grzelak M. J. Rasoloson D. (2017). Precision genome editing using synthesis-dependent repair of Cas9-induced DNA breaks. *Proc. Natl. Acad. Sci. U. S. A.* 114 E10745–E10754. 29183983 10.1073/pnas.1711979114 PMC5740635

[B160] Pannunzio N. R. Watanabe G. Lieber M. R. (2018). Nonhomologous DNA end-joining for repair of DNA double-strand breaks. *J. Biol. Chem.* 293 10512–10523. 10.1074/jbc.tm117.000374 29247009 PMC6036208

[B161] Park K.-E. Kaucher A. V. Powell A. Waqas M. S. Sandmaier S. E. Oatley M. J. (2017). Generation of germline ablated male pigs by CRISPR/Cas9 editing of the NANOS2 gene. *Sci. Rep.* 7:40176. 10.1038/srep40176 PMC5223215 28071690

[B162] Pawelczak K. S. Gavande N. S. Vandervere-Carozza P. S. Turchi J. J. (2018). Modulating DNA repair pathways to improve precision genome engineering. *ACS Chem. Biol.* 13 389–396. 10.1021/acschembio.7b00777 29210569

[B163] Peng J. Wang Y. Jiang J. Zhou X. Song L. Wang L. (2015). Production of human albumin in pigs through CRISPR/Cas9-mediated knockin of human cDNA into swine albumin locus in the zygotes. *Sci. Rep.* 5:16705. 10.1038/srep16705 PMC4642324 26560187

[B164] Perisse I. V. Fan Z. Van Wettere A. Wang Z. Harris A. White K. (2020). 132 Introduction of F508del human mutation into the CFTR gene of sheep fetal fibroblasts using CRISPR/Cas9 ribonucleoprotein. *Reprod. Fertil. Dev.* 32 192–193. 10.1071/rdv32n2ab132

[B165] Perota A. Lagutina I. Duchi R. Zanfrini E. Lazzari G. Judor J. P. (2019). Generation of cattle knockout for galactose-α1, 3-galactose and N-glycolylneuraminic acid antigens. *Xenotransplantation* 26:e12524. 10.1111/xen.12524 PMC6852128 31115108

[B166] Petersen B. Frenzel A. Lucas-Hahn A. Herrmann D. Hassel P. Klein S. (2016). Efficient production of biallelic GGTA 1 knockout pigs by cytoplasmic microinjection of CRISPR/Cas9 into zygotes. *Xenotransplantation* 23 338–346. 10.1111/xen.12258 27610605

[B167] Phelps C. J. Koike C. Vaught T. D. Boone J. Wells K. D. Chen S. H. (2003). Production of alpha 1,3-galactosyltransferase-deficient pigs. *Science* 299 411–414. 10.1126/science.1078942 12493821 PMC3154759

[B168] Polejaeva I. A. Chen S.-H. Vaught T. D. Page R. L. Mullins J. Ball S. (2000). Cloned pigs produced by nuclear transfer from adult somatic cells. *Nature* 407 86–90. 10.1038/35024082 10993078

[B169] Polejaeva I. A. Rutigliano H. M. Wells K. D. (2016). Livestock in biomedical research: history, current status and future prospective. *Reprod. Fertil. Dev.* 28 112–124. 10.1071/rd15343 27062879

[B170] Polejaeva I. Campbell K. (2000). New advances in somatic cell nuclear transfer: application in transgenesis. *Theriogenology* 53 117–126. 10.1016/s0093-691x(99)00245-9 10735067

[B171] Prusiner S. B. (1998). Prions. *Proc. Natl. Acad. Sci. U. S. A.* 95 13363–13383. 9811807 10.1073/pnas.95.23.13363 PMC33918

[B172] Pursel V. G. Rexroad C. E. (1993). Status of research with transgenic farm-animals. *J. Anim. Sci.* 71 10–19. 10.2527/1993.71suppl_310x 8505265

[B173] Qomi S. B. Asghari A. Mojarrad M. (2019). An overview of the CRISPR-based genomic-and epigenome-editing system: function, applications, and challenges. *Adv. Biomed. Res.* 8:49 10.4103/abr.abr_41_19 PMC6712897 31516887

[B174] Rees H. A. Liu D. R. (2018). Base editing: precision chemistry on the genome and transcriptome of living cells. *Nat. Rev. Genet.* 19 770–788. 10.1038/s41576-018-0059-1 30323312 PMC6535181

[B175] Ren J. Yu D. Fu R. An P. Sun R. Wang Z. (2020). IL2RG-deficient minipigs generated via CRISPR/Cas9 technology support the growth of human melanoma-derived tumours. *Cell Proliferation* 53:e12863. 10.1111/cpr.12863 PMC7574875 32871045

[B176] Renaud J.-B. Boix C. Charpentier M. De Cian A. Cochennec J. Duvernois-Berthet E. (2016). Improved genome editing efficiency and flexibility using modified oligonucleotides with TALEN and CRISPR-Cas9 nucleases. *Cell Rep.* 14 2263–2272. 10.1016/j.celrep.2016.02.018 26923600

[B177] Reyes L. M. Estrada J. L. Wang Z. Y. Blosser R. J. Smith R. F. Sidner R. A. (2014). Creating class I MHC–null pigs using guide RNA and the Cas9 endonuclease. *J. Immunol.* 193 5751–5757. 10.4049/jimmunol.1402059 25339675 PMC5922270

[B178] Reynolds L. P. Ireland J. J. Caton J. S. Bauman D. E. Davis T. A. (2009). Commentary on domestic animals in agricultural and biomedical research: an endangered enterprise. *J. Nutr.* 139 427–428. 10.3945/jn.108.103564 19158219 PMC3314500

[B179] Richt J. A. Kasinathan P. Hamir A. N. Castilla J. Sathiyaseelan T. Vargas F. (2007). Production of cattle lacking prion protein. *Nat. Biotechnol.* 25 132–138. 10.1038/nbt1271 17195841 PMC2813193

[B180] Richter M. F. Zhao K. T. Eton E. Lapinaite A. Newby G. A. Thuronyi B. W. (2020). Phage-assisted evolution of an adenine base editor with improved Cas domain compatibility and activity. *Nat. Biotechnol.* 38 883–891. 10.1038/s41587-020-0453-z 32433547 PMC7357821

[B181] Riordan S. M. Heruth D. P. Zhang L. Q. Ye S. Q. (2015). Application of CRISPR/Cas9 for biomedical discoveries. *Cell Biosci.* 5:33. 10.1186/s13578-015-0027-9 PMC4487574 26137216

[B182] Robert F. Barbeau M. Éthier S. Dostie J. Pelletier J. (2015). Pharmacological inhibition of DNA-PK stimulates Cas9-mediated genome editing. *Genome Med.* 7:93. 10.1186/s13073-015-0215-6 PMC4550049 26307031

[B183] Roberts S. B. Goetz F. W. (2003). Myostatin protein and RNA transcript levels in adult and developing brook trout. *Mol. Cell. Endocrinol.* 210 9–20. 10.1016/j.mce.2003.09.002 14615056

[B184] Rosenthal N. Brown S. (2007). The mouse ascending: perspectives for human-disease models. *Nat. Cell Biol.* 9 993–999. 10.1038/ncb437 17762889

[B185] Roth J. A. Tuggle C. K. (2015). Livestock models in translational medicine. *ILAR J.* 56 1–6. 10.1093/ilar/ilv011 25991694

[B186] Ruan J. Li H. Xu K. Wu T. Wei J. Zhou R. (2015). Highly efficient CRISPR/Cas9-mediated transgene knockin at the H11 locus in pigs. *Sci. Rep.* 5:14253. 10.1038/srep14253 PMC4585612 26381350

[B187] Sake H. J. Frenzel A. Lucas-Hahn A. Nowak-Imialek M. Hassel P. Hadeler K. G. (2019). Possible detrimental effects of beta-2-microglobulin knockout in pigs. *Xenotransplantation* 26:e12525. 10.1111/xen.12525 31119817

[B188] Sakuma T. Nakade S. Sakane Y. Suzuki K.-I. T. Yamamoto T. (2016). MMEJ-assisted gene knock-in using TALENs and CRISPR-Cas9 with the PITCh systems. *Nat. Protoc.* 11 118–133. 10.1038/nprot.2015.140 26678082

[B189] Schnieke A. E. Kind A. J. Ritchie W. A. Mycock K. Scott A. R. Ritchie M. (1997). Human factor IX transgenic sheep produced by transfer of nuclei from transfected fetal fibroblasts. *Science* 278 2130–2133. 10.1126/science.278.5346.2130 9405350

[B190] Schuelke M. Wagner K. R. Stolz L. E. Hubner C. Riebel T. Komen W. (2004). Myostatin mutation associated with gross muscle hypertrophy in a child. *N. Engl. J. Med.* 350 2682–2688. 10.1056/nejmoa040933 15215484

[B191] Schuster F. Aldag P. Frenzel A. Hadeler K.-G. Lucas-Hahn A. Niemann H. (2020). CRISPR/Cas12a mediated knock-in of the polled celtic variant to produce a polled genotype in dairy cattle. *Sci. Rep.* 10:13570. 10.1038/s41598-020-70531-y PMC7419524 32782385

[B192] Sehnal D. Rose A. S. Koča J. Burley S. K. Velankar S. (2018). “Mol^∗^: towards a common library and tools for web molecular graphics,” in *Proceedings of the Workshop on Molecular Graphics and Visual Analysis of Molecular Data*, eds Byska J. Krone M. Sommer B. (Brno: Eurographics Association).

[B193] Seki A. Rutz S. (2018). Optimized RNP transfection for highly efficient CRISPR/Cas9-mediated gene knockout in primary T cells. *J. Exp. Med.* 215 985–997. 10.1084/jem.20171626 29436394 PMC5839763

[B194] Sheets T. P. Park C. H. Park K. E. Powell A. Donovan D. M. Telugu B. P. (2016). Somatic cell nuclear transfer followed by CRIPSR/Cas9 microinjection results in highly efficient genome editing in cloned pigs. *Intl. J. Mol. Sci.* 17:2031. 10.3390/ijms17122031 27918485 PMC5187831

[B195] Shi M. Kawabe Y. Ito A. Kamihira M. (2020). Targeted knock-in into the OVA locus of chicken cells using CRISPR/Cas9 system with homology-independent targeted integration. *J. Biosci. Bioeng.* 129 363–370. 10.1016/j.jbiosc.2019.09.011 31594694

[B196] Sommer D. Peters A. E. Wirtz T. Mai M. Ackermann J. Thabet Y. (2014). Efficient genome engineering by targeted homologous recombination in mouse embryos using transcription activator-like effector nucleases. *Nat. Commun.* 5:3045. 10.1038/ncomms4045 24413636

[B197] Song J. Yang D. Xu J. Zhu T. Chen Y. E. Zhang J. (2016). RS-1 enhances CRISPR/Cas9-and TALEN-mediated knock-in efficiency. *Nat. Commun.* 7:10548. 10.1038/ncomms10548 PMC4738357 26817820

[B198] Stoian A. Rowland R. R. Petrovan V. Sheahan M. Samuel M. S. Whitworth K. M. (2020). The use of cells from ANPEP knockout pigs to evaluate the role of aminopeptidase N (APN) as a receptor for porcine deltacoronavirus (PDCoV). *Virology* 541 136–140. 10.1016/j.virol.2019.12.007 32056711 PMC7112016

[B199] Sun Z. Wang M. Han S. Ma S. Zou Z. Ding F. (2018). Production of hypoallergenic milk from DNA-free beta-lactoglobulin (BLG) gene knockout cow using zinc-finger nucleases mRNA. *Sci. Rep.* 8:15430. 10.1038/s41598-018-32024-x PMC6194018 30337546

[B200] Suzuki K. Belmonte J. C. I. (2018). In vivo genome editing via the HITI method as a tool for gene therapy. *J. Hum. Genet.* 63 157–164. 10.1038/s10038-017-0352-4 29215090

[B201] Suzuki K. Tsunekawa Y. Hernandez-Benitez R. Wu J. Zhu J. Kim E. J. (2016). In vivo genome editing via CRISPR/Cas9 mediated homology-independent targeted integration. *Nature* 540 144–149. 27851729 10.1038/nature20565 PMC5331785

[B202] Tan W. Carlson D. F. Lancto C. A. Garbe J. R. Webster D. A. Hackett P. B. (2013). Efficient nonmeiotic allele introgression in livestock using custom endonucleases. *Proc. Natl. Acad. Sci. U. S. A.* 110 16526–16531. 10.1073/pnas.1310478110 24014591 PMC3799378

[B203] Tanihara F. Hirata M. Nguyen N. T. Le Q. A. Hirano T. Takemoto T. (2018). Generation of a TP53-modified porcine cancer model by CRISPR/Cas9-mediated gene modification in porcine zygotes via electroporation. *PLoS One* 13:e0206360. 10.1371/journal.pone.0206360 30352075 PMC6198999

[B204] Tanihara F. Hirata M. Nguyen N. T. Le Q. A. Wittayarat M. Fahrudin M. (2019). Generation of CD163-edited pig via electroporation of the CRISPR/Cas9 system into porcine in vitro-fertilized zygotes. *Anim. Biotechnol.* 10.1080/10495398.2019.1668801 Online ahead of print. 31558095

[B205] Tanihara F. Hirata M. Nguyen N. T. Sawamoto O. Kikuchi T. Doi M. (2020). Efficient generation of GGTA1-deficient pigs by electroporation of the CRISPR/Cas9 system into in vitro-fertilized zygotes. *BMC Biotechnol.* 20:40. 10.1186/s12896-020-00638-7 PMC7436961 32811500

[B206] Tilman D. Balzer C. Hill J. Befort B. L. (2011). Global food demand and the sustainable intensification of agriculture. *Proc. Natl. Acad. Sci. U. S. A.* 108 20260–20264. 22106295 10.1073/pnas.1116437108 PMC3250154

[B207] Tsai S. Q. Zheng Z. Nguyen N. T. Liebers M. Topkar V. V. Thapar V. (2015). GUIDE-seq enables genome-wide profiling of off-target cleavage by CRISPR-Cas nucleases. *Nat. Biotechnol.* 33 187–197. 10.1038/nbt.3117 25513782 PMC4320685

[B208] Tu C.-F. Chuang C.-K. Hsiao K.-H. Chen C.-H. Chen C.-M. Peng S.-H. (2019). Lessening of porcine epidemic diarrhoea virus susceptibility in piglets after editing of the CMP-N-glycolylneuraminic acid hydroxylase gene with CRISPR/Cas9 to nullify N-glycolylneuraminic acid expression. *PLoS One* 14:e0217236. 10.1371/journal.pone.0217236 31141512 PMC6541307

[B209] Tycko J. Myer V. E. Hsu P. D. (2016). Methods for optimizing CRISPR-Cas9 genome editing specificity. *Mol. Cell.* 63 355–370. 10.1016/j.molcel.2016.07.004 27494557 PMC4976696

[B210] United Nations (2020). *World Population Prospects. The 2017 Revision.* Available online at: https://www.un.org/esa/population/. (accessed September 29, 2020)

[B211] Urnov F. D. Rebar E. J. Holmes M. C. Zhang H. S. Gregory P. D. (2010). Genome editing with engineered zinc finger nucleases. *Nat. Rev. Genet.* 11 636–646. 10.1038/nrg2842 20717154

[B212] Van der Velden J. Snibson K. J. (2011). Airway disease: the use of large animal models for drug discovery. *Pulm. Pharmacol. Ther.* 24 525–532. 10.1016/j.pupt.2011.02.001 21356324

[B213] Vartak S. V. Raghavan S. C. (2015). Inhibition of nonhomologous end joining to increase the specificity of CRISPR/Cas9 genome editing. *FEBS J.* 282 4289–4294. 10.1111/febs.13416 26290158

[B214] Vilarino M. Rashid S. T. Suchy F. P. Mcnabb B. R. Van Der Meulen T. Fine E. J. (2017). CRISPR/Cas9 microinjection in oocytes disables pancreas development in sheep. *Sci. Rep.* 7:17472. 10.1038/s41598-017-17805-0 PMC5727233 29234093

[B215] Wall R. J. (1996). Transgenic livestock: progress and prospects for the future. *Theriogenology* 45 57–68. 10.1016/0093-691x(95)00355-c

[B216] Wang J. Liu M. Zhao L. Li Y. Zhang M. Jin Y. (2019). Disabling of nephrogenesis in porcine embryos via CRISPR/Cas9-mediated SIX1 and SIX4 gene targeting. *Xenotransplantation* 26:e12484. 10.1111/xen.12484 30623494

[B217] Wang K. Ouyang H. Xie Z. Yao C. Guo N. Li M. (2015). Efficient generation of myostatin mutations in pigs using the CRISPR/Cas9 system. *Sci. Rep.* 5:16623. 10.1038/srep16623 PMC4643223 26564781

[B218] Wang K. Tang X. Liu Y. Xie Z. Zou X. Li M. (2016). Efficient generation of orthologous point mutations in pigs via CRISPR-assisted ssODN-mediated homology-directed repair. *Mol. Ther. Nucleic Acids* 5:e396. 10.1038/mtna.2016.101 27898095 PMC5155319

[B219] Wang K. Tang X. Xie Z. Zou X. Li M. Yuan H. (2017). CRISPR/Cas9-mediated knockout of myostatin in Chinese indigenous Erhualian pigs. *Transgenic Res.* 26 799–805. 10.1007/s11248-017-0044-z 28993973

[B220] Wang X. Cao C. Huang J. Yao J. Hai T. Zheng Q. (2016a). One-step generation of triple gene-targeted pigs using CRISPR/Cas9 system. *Sci. Rep.* 6:20620. 10.1038/srep20620 PMC4746670 26857844

[B221] Wang X. Niu Y. Zhou J. Yu H. Kou Q. Lei A. (2016b). Multiplex gene editing via CRISPR/Cas9 exhibits desirable muscle hypertrophy without detectable off-target effects in sheep. *Sci. Rep.* 6:32271. 10.1038/srep32271 PMC4999810 27562433

[B222] Wang X. Yu H. Lei A. Zhou J. Zeng W. Zhu H. (2015a). Generation of gene-modified goats targeting MSTN and FGF5 via zygote injection of CRISPR/Cas9 system. *Sci. Rep.* 5:13878. 10.1038/srep13878 PMC4564737 26354037

[B223] Wang X. Zhou J. Cao C. Huang J. Hai T. Wang Y. (2015b). Efficient CRISPR/Cas9-mediated biallelic gene disruption and site-specific knockin after rapid selection of highly active sgRNAs in pigs. *Sci. Rep.* 5:13348. 10.1038/srep13348 PMC4543986 26293209

[B224] Wang Y. Du Y. Shen B. Zhou X. Li J. Liu Y. (2015). Efficient generation of gene-modified pigs via injection of zygote with Cas9/sgRNA. *Sci. Rep.* 5:8256. 10.1038/srep08256 PMC4317696 25653176

[B225] Weber T. Graf R. Sommermann T. Petsch K. Sack U. Volchkov P. (2016). Efficient generation of Rosa26 knock-in mice using CRISPR/Cas9 in C57BL/6 zygotes. *BMC Biotechnol.* 16:4. 10.1186/s12896-016-0234-4 PMC4715285 26772810

[B226] Whitelaw C. B. Sheets T. P. Lillico S. G. Telugu B. P. (2016). Engineering large animal models of human disease. *J. Pathol.* 238 247–256. 26414877 10.1002/path.4648 PMC4737318

[B227] Whittemore L. A. Song K. Li X. Aghajanian J. Davies M. Girgenrath S. (2003). Inhibition of myostatin in adult mice increases skeletal muscle mass and strength. *Biochem. Biophys. Res. Commun.* 300 965–971. 10.1016/s0006-291x(02)02953-4 12559968

[B228] Whitworth K. M. Benne J. A. Spate L. D. Murphy S. L. Samuel M. S. Murphy C. N. (2017). Zygote injection of CRISPR/Cas9 RNA successfully modifies the target gene without delaying blastocyst development or altering the sex ratio in pigs. *Transgenic Res.* 26 97–107. 10.1007/s11248-016-9989-6 27744533 PMC5247313

[B229] Whitworth K. M. Lee K. Benne J. A. Beaton B. P. Spate L. D. Murphy S. L. (2014). Use of the CRISPR/Cas9 system to produce genetically engineered pigs from in vitro-derived oocytes and embryos. *Biol. Reprod.* 91 71–13. 25100712 10.1095/biolreprod.114.121723 PMC4435063

[B230] Whitworth K. M. Rowland R. R. R. Ewen C. L. Trible B. R. Kerrigan M. A. Cino-Ozuna A. G. (2016). Gene-edited pigs are protected from porcine reproductive and respiratory syndrome virus. *Nat. Biotechnol.* 34 20–22. 10.1038/nbt.3434 26641533

[B231] Whitworth K. M. Rowland R. R. Petrovan V. Sheahan M. Cino-Ozuna A. G. Fang Y. (2019). Resistance to coronavirus infection in amino peptidase N-deficient pigs. *Transgenic Res.* 28 21–32. 10.1007/s11248-018-0100-3 30315482 PMC6353812

[B232] Wieland S. Schatt M. D. Rusconi S. (1990). Role of TATA-element in transcription from glucocorticoid receptor-responsive model promoters. *Nucleic Acids Res.* 18 5113–5118. 10.1093/nar/18.17.5113 2402438 PMC332131

[B233] Williams D. K. Pinzón C. Huggins S. Pryor J. H. Falck A. Herman F. (2018). Genetic engineering a large animal model of human hypophosphatasia in sheep. *Sci. Rep.* 8:16945. 10.1038/s41598-018-35079-y PMC6240114 30446691

[B234] Wilmut I. Clark A. J. (1991). Basic techniques for transgenesis. *J Reprod. Fertil. Suppl.* 43 265–275. 1843345

[B235] Wilmut I. Schnieke A. E. Mcwhir J. Kind A. J. Campbell K. H. (1997). Viable offspring derived from fetal and adult mammalian cells. *Nature* 385 810–813. 10.1038/385810a0 9039911

[B236] Woods G. L. White K. L. Vanderwall D. K. Li G.-P. Aston K. I. Bunch T. D. (2003). A mule cloned from fetal cells by nuclear transfer. *Science* 301:1063. 10.1126/science.1086743 12775846

[B237] Wu J. Vilarino M. Suzuki K. Okamura D. Bogliotti Y. S. Park I. (2017). CRISPR-Cas9 mediated one-step disabling of pancreatogenesis in pigs. *Sci. Rep.* 7:10487. 10.1038/s41598-017-08596-5 PMC5585254 28874671

[B238] Wu M. Wei C. Lian Z. Liu R. Zhu C. Wang H. (2016). Rosa26-targeted sheep gene knock-in via CRISPR-Cas9 system. *Sci. Rep.* 6:24360. 10.1038/srep24360 PMC4827023 27063570

[B239] Xiang G. Ren J. Hai T. Fu R. Yu D. Wang J. (2018). Editing porcine IGF2 regulatory element improved meat production in Chinese Bama pigs. *Cell. Mol. Life Sci.* 75 4619–4628. 10.1007/s00018-018-2917-6 30259067 PMC11105340

[B240] Xie J. Ge W. Li N. Liu Q. Chen F. Yang X. (2019). Efficient base editing for multiple genes and loci in pigs using base editors. *Nat. Commun.* 10:2852. 10.1038/s41467-019-10421-8 PMC6599043 31253764

[B241] Xie Z. Jiao H. Xiao H. Jiang Y. Liu Z. Qi C. (2020). Generation of pRSAD2 gene knock-in pig via CRISPR/Cas9 technology. *Antivir. Res.* 174:104696. 10.1016/j.antiviral.2019.104696 31862502

[B242] Xie Z. Pang D. Wang K. Li M. Guo N. Yuan H. (2017). Optimization of a CRISPR/Cas9-mediated knock-in strategy at the porcine Rosa26 locus in porcine foetal fibroblasts. *Sci. Rep.* 7:3036. 10.1038/s41598-017-02785-y PMC5465212 28596588

[B243] Xu K. Zhou Y. Mu Y. Liu Z. Hou S. Xiong Y. (2020). CD163 and pAPN double-knockout pigs are resistant to PRRSV and TGEV and exhibit decreased susceptibility to PDCoV while maintaining normal production performance. *Elife* 9:e57132. 10.7554/eLife.57132 PMC7467724 32876563

[B244] Yan J. Cirincione A. Adamson B. (2020). Prime editing: precision genome editing by reverse transcription. *Mol. Cell.* 77 210–212. 10.1016/j.molcel.2019.12.016 31951546

[B245] Yan S. Tu Z. Liu Z. Fan N. Yang H. Yang S. (2018). A huntingtin knockin pig model recapitulates features of selective neurodegeneration in Huntington’s disease. *Cell* 173 989–1002.e13. 29606351 10.1016/j.cell.2018.03.005 PMC5935586

[B246] Yang H. Zhang J. Zhang X. Shi J. Pan Y. Zhou R. (2018). CD163 knockout pigs are fully resistant to highly pathogenic porcine reproductive and respiratory syndrome virus. *Antivir. Res.* 151 63–70. 10.1016/j.antiviral.2018.01.004 29337166

[B247] Yang X. (2015). Applications of CRISPR-Cas9 mediated genome engineering. *Mil. Med. Res.* 2:11. 10.1186/s40779-015-0038-1 PMC4433013 25984354

[B248] Yao X. Wang X. Hu X. Liu Z. Liu J. Zhou H. (2017a). Homology-mediated end joining-based targeted integration using CRISPR/Cas9. *Cell Res.* 27 801–814. 10.1038/cr.2017.76 28524166 PMC5518881

[B249] Yao X. Wang X. Liu J. Hu X. Shi L. Shen X. (2017b). CRISPR/Cas9–Mediated precise targeted integration in vivo using a double cut donor with short homology arms. *EBioMedicine* 20 19–26. 10.1016/j.ebiom.2017.05.015 28527830 PMC5478232

[B250] Yasue A. Kono H. Habuta M. Bando T. Sato K. Inoue J. (2017). Relationship between somatic mosaicism of Pax6 mutation and variable developmental eye abnormalities—an analysis of CRISPR genome-edited mouse embryos. *Sci. Rep.* 7:53. 10.1038/s41598-017-00088-w PMC5428340 28246397

[B251] Yeh C. D. Richardson C. D. Corn J. E. (2019). Advances in genome editing through control of DNA repair pathways. *Nat. Cell Biol.* 21 1468–1478. 10.1038/s41556-019-0425-z 31792376

[B252] Yi D. Zhou S.-W. Qiang D. Bei C. Zhao X.-E. Zhong S. (2020). The CRISPR/Cas9 induces large genomic fragment deletions of MSTN and phenotypic changes in sheep. *J. Integr. Agricu.* 19 1065–1073. 10.1016/s2095-3119(19)62853-4

[B253] Yoshimi K. Kunihiro Y. Kaneko T. Nagahora H. Voigt B. Mashimo T. (2016). ssODN-mediated knock-in with CRISPR-Cas for large genomic regions in zygotes. *Nat. Commun.* 7:10431. 10.1038/ncomms10431 PMC4736110 26786405

[B254] Yu C. Liu Y. Ma T. Liu K. Xu S. Zhang Y. (2015). Small molecules enhance CRISPR genome editing in pluripotent stem cells. *Cell Stem Cell* 16 142–147. 10.1016/j.stem.2015.01.003 25658371 PMC4461869

[B255] Yu H.-H. Zhao H. Qing Y.-B. Pan W.-R. Jia B.-Y. Zhao H.-Y. (2016). Porcine zygote injection with Cas9/sgRNA results in DMD-modified pig with muscle dystrophy. *Intl. J. Mol. Sci.* 17:1668. 10.3390/ijms17101668 27735844 PMC5085701

[B256] Yu M. Sun X. Tyler S. R. Liang B. Swatek A. M. Lynch T. J. (2019). Highly efficient transgenesis in ferrets using CRISPR/Cas9-mediated homology-independent insertion at the ROSA26 locus. *Sci. Rep.* 9:1971. 10.1038/s41598-018-37192-4 PMC6374392 30760763

[B257] Yu Y. Guo Y. Tian Q. Lan Y. Yeh H. Zhang M. (2020). An efficient gene knock-in strategy using 5′-modified double-stranded DNA donors with short homology arms. *Nat. Chem. Biol.* 16 387–390. 10.1038/s41589-019-0432-1 31873222 PMC7085973

[B258] Yugo D. M. Heffron C. L. Ryu J. Uh K. Subramaniam S. Matzinger S. R. (2018). Infection dynamics of hepatitis E virus in wild-type and immunoglobulin heavy chain knockout JH-/- gnotobiotic piglets. *J. Virol.* 92 e010208–e010218. 10.1128/JVI.01208-18 PMC6189505 30111571

[B259] Zafra M. P. Schatoff E. M. Katti A. Foronda M. Breinig M. Schweitzer A. Y. (2018). Optimized base editors enable efficient editing in cells, organoids and mice. *Nat. Biotechnol.* 36 888–893. 10.1038/nbt.4194 29969439 PMC6130889

[B260] Zhang J. Cui M. L. Nie Y. W. Dai B. Li F. R. Liu D. J. (2018). CRISPR/Cas9-mediated specific integration of fat-1 at the goat MSTN locus. *FEBS J.* 285 2828–2839. 10.1111/febs.14520 29802684

[B261] Zhang R. Li Y. Jia K. Xu X. Li Y. Zhao Y. (2020). Crosstalk between androgen and Wnt/β-catenin leads to changes of wool density in FGF5-knockout sheep. *Cell Death Dis.* 11:407. 10.1038/s41419-020-2622-x PMC7260202 32472005

[B262] Zhang R. Wang Y. Chen L. Wang R. Li C. Li X. (2018). Reducing immunoreactivity of porcine bioprosthetic heart valves by genetically-deleting three major glycan antigens, GGTA1/β4GalNT2/CMAH. *Acta Biomater.* 72 196–205. 10.1016/j.actbio.2018.03.055 29631050

[B263] Zhang W. Wang G. Wang Y. Jin Y. Zhao L. Xiong Q. (2017). Generation of complement protein C3 deficient pigs by CRISPR/Cas9-mediated gene targeting. *Sci. Rep.* 7:5009. 10.1038/s41598-017-05400-2 PMC5503937 28694465

[B264] Zhang X. Li W. Liu C. Peng X. Lin J. He S. (2017). Alteration of sheep coat color pattern by disruption of ASIP gene via CRISPR Cas9. *Sci. Rep.* 7:8149. 10.1038/s41598-017-08636-0 PMC5557758 28811591

[B265] Zhang X.-H. Tee L. Y. Wang X.-G. Huang Q.-S. Yang S.-H. (2015). Off-target effects in CRISPR/Cas9-mediated genome engineering. *Mol. Ther. Nucleic Acids* 4:e264. 10.1038/mtna.2015.37 26575098 PMC4877446

[B266] Zhang Y. Wang Y. Yulin B. Tang B. Wang M. Zhang C. (2019). CRISPR/Cas9-mediated sheep MSTN gene knockout and promote sSMSCs differentiation. *J. Cell. Biochem.* 120 1794–1806. 10.1002/jcb.27474 30242885

[B267] Zhao X. Wei C. Li J. Xing P. Li J. Zheng S. (2017). Cell cycle-dependent control of homologous recombination. *Acta Biochim. Biophys. Sin.* 49 655–668. 10.1093/abbs/gmx055 28541389

[B268] Zheng Q. Lin J. Huang J. Zhang H. Zhang R. Zhang X. (2017). Reconstitution of UCP1 using CRISPR/Cas9 in the white adipose tissue of pigs decreases fat deposition and improves thermogenic capacity. *Proc. Natl. Acad. Sci. U. S. A.* 114 E9474–E9482. 29078316 10.1073/pnas.1707853114 PMC5692550

[B269] Zhong H. Chen Y. Li Y. Chen R. Mardon G. (2015). CRISPR-engineered mosaicism rapidly reveals that loss of Kcnj13 function in mice mimics human disease phenotypes. *Sci. Rep.* 5:8366. 10.1038/srep08366 PMC4322368 25666713

[B270] Zhou S. Cai B. He C. Wang Y. Ding Q. Liu J. (2019). Programmable base editing of the sheep genome revealed no genome-wide off-target mutations. *Front. Genet.* 10:215. 10.3389/fgene.2019.00215 PMC6428697 30930940

[B271] Zhou S. Yu H. Zhao X. Cai B. Ding Q. Huang Y. (2018). Generation of gene-edited sheep with a defined Booroola fecundity gene (FecBB) mutation in bone morphogenetic protein receptor type 1B (BMPR1B) via clustered regularly interspaced short palindromic repeat (CRISPR)/CRISPR-associated (Cas) 9. *Reprod. Fertil. Dev.* 30 1616–1621. 10.1071/rd18086 31039970

[B272] Zhou W. Wan Y. Guo R. Deng M. Deng K. Wang Z. (2017). Generation of beta-lactoglobulin knock-out goats using CRISPR/Cas9. *PLoS One* 12:e0186056. 10.1371/journal.pone.0186056 29016691 PMC5634636

[B273] Zhou X. Xin J. Fan N. Zou Q. Huang J. Ouyang Z. (2015). Generation of CRISPR/Cas9-mediated gene-targeted pigs via somatic cell nuclear transfer. *Cell Mol. Life. Sci.* 72 1175–1184. 10.1007/s00018-014-1744-7 25274063 PMC11113635

[B274] Zhu X. Wei Y. Zhan Q. Yan A. Feng J. Liu L. (2020). CRISPR/Cas9-Mediated Biallelic Knockout of IRX3 Reduces the Production and Survival of Somatic Cell-Cloned Bama Minipigs. *Animals* 10:501. 10.3390/ani10030501 32192102 PMC7142520

[B275] Zhu X.-X. Zhong Y.-Z. Ge Y.-W. Lu K.-H. Lu S.-S. (2018). CRISPR/Cas9-mediated generation of Guangxi Bama minipigs harboring three mutations in α-synuclein causing Parkinson’s disease. *Sci. Rep.* 8:12420. 10.1038/s41598-018-30436-3 PMC6102220 30127453

[B276] Zou X. Ouyang H. Yu T. Chen X. Pang D. Tang X. (2019). Preparation of a new type 2 diabetic miniature pig model via the CRISPR/Cas9 system. *Cell Death Dis.* 10:823. 10.1038/s41419-019-2056-5 PMC6817862 31659151

[B277] Zou Y. Li Z. Zou Y. Hao H. Li N. Li Q. (2018). An FBXO40 knockout generated by CRISPR/Cas9 causes muscle hypertrophy in pigs without detectable pathological effects. *Biochem. Biophys. Res. Commun.* 498 940–945. 10.1016/j.bbrc.2018.03.085 29545179

[B278] Zou Y.-L. Li Z.-Y. Zou Y.-J. Hao H.-Y. Hu J.-X. Li N. (2019). Generation of pigs with a Belgian Blue mutation in MSTN using CRISPR/Cpf1-assisted ssODN-mediated homologous recombination. *J. Integr. Agricu.* 18 1329–1336. 10.1016/s2095-3119(19)62694-8

[B279] Zuccaro M. V. Xu J. Mitchell C. Marin D. Zimmerman R. Rana B. (2020). Allele-specific chromosome removal after Cas9 cleavage in human embryos. *Cell* S009 31381–31389. 10.1016/j.cell.2020.10.025 33125898

[B280] Zuo E. Sun Y. Wei W. Yuan T. Ying W. Sun H. (2019). Cytosine base editor generates substantial off-target single-nucleotide variants in mouse embryos. *Science* 364 289–292. 30819928 10.1126/science.aav9973 PMC7301308

[B281] Zwaka T. P. Thomson J. A. (2009). “Chapter 46 - homologous recombination in human embryonic stem cells,” in *Essentials of Stem Cell Biology (Second Edition)*, eds Lanza R. Gearhart J. Hogan B. Melton D. Pedersen R. Thomas E. D. (San Diego: Academic Press), 417–422. 10.1016/b978-0-12-374729-7.00046-9

